# Tip60‐Mediated USP22 Lactylation Drives Glutamine Metabolic Reprogramming via SLC25A5 in TNBC

**DOI:** 10.1002/advs.78486

**Published:** 2026-10-26

**Authors:** Fang Zhen, Hongyi Wang, Ningzhi Zhang, Le Wang, Huiyuan Tian, Luxi Kang, Zhanpeng Liu, Jing Hu

**Affiliations:** ^1^ Department of Breast Medical Oncology Harbin Medical University Cancer Hospital Harbin Medical University Harbin Heilongjiang Province China

**Keywords:** glutamine metabolism, macrophages, metabolic reprogramming, triple‐negative breast cancer, USP22

## Abstract

Glutamine metabolism promotes breast cancer progression and metastasis, though the underlying mechanisms are not fully understood. In this study, we identified a significant positive correlation between glutamine metabolism and ubiquitin‐specific protease 22 (USP22)expression in breast cancer patients. USP22 drives glutamine reprogramming to promote lactate secretion, thereby supporting triple‐negative breast cancer (TNBC) growth and survival. This process involves the lactyltransferase Tat‐interactive protein, 60 kDa (Tip60), which catalyzes K134 lactylation of USP22. This post‐translational modification (PTM) stabilizes USP22 protein levels and facilitates its mitochondrial location. Moreover, USP22 stabilizes the solute carrier family 25 member 5 (SLC25A5) through its de‐ubiquitinase activity. Clinically, increased expression levels of both USP22 and SLC25A5 are significantly associated with a poor prognosis in patients with TNBC. Furthermore, USP22 regulates glutamine metabolism to promote α‐ketoglutarate (α‐KG) secretion, thereby activating M2 macrophages and suppressing the anti‐tumor immune response. Importantly, targeting USP22 synergized with CB‐839 in TNBC xenograft models. These findings underscore USP22 as a promising therapeutic target for TNBC.

## Graphical abstract

Tip60‐mediated USP22 K134 lactylation, stabilizing SLC25A5 to enhance glutamine metabolism and α‐KG secretion. The secreted α‐KG drives GPR91‐dependent M2 macrophage polarization, promoting triple‐negative breast cancer progression. Targeting this axis synergizes with CB‐839, offering a promising therapeutic strategy for TNBC.

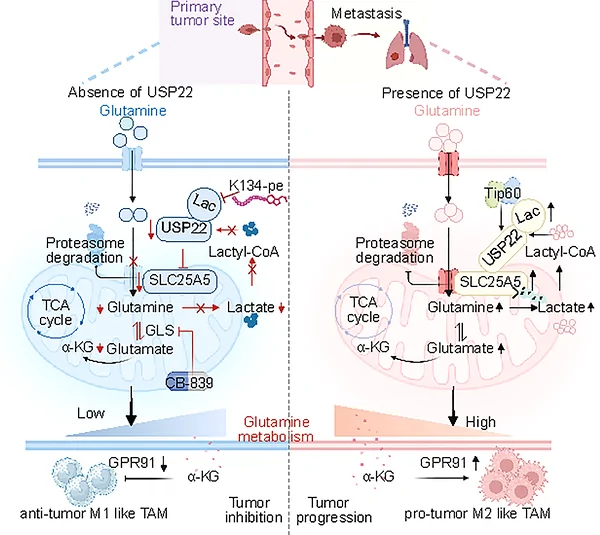

## 1 Introduction

Breast cancer is the most prevalent malignancy and the leading cause of cancer‐related death among women, accounting for approximately one‐third of new female cancer cases in 2022 [1]. TNBC is associated with poorer prognosis, higher recurrence rates, and greater metastatic potential than other breast cancer subtypes [2]. However, therapeutic options for TNBC remain limited. A deeper understanding of the molecular pathways underlying TNBC is urgently needed to improve chemotherapy responsiveness.

Metabolic reprogramming is a hallmark of cancer. To sustain continuous proliferation, tumor cells rewire metabolic networks to meet energy and biosynthetic demands while maintaining tumorigenicity [3]. Glutamine serves as a crucial metabolic substrate [4], supplying carbon and nitrogen sources for the synthesis of nucleotides, nonessential amino acids, and lipids. Glutamine metabolism is a central pathway in cancer that drives tumor cell proliferation, invasion, and metastasis [5, 6]. Glutamine is further metabolized by GLS and GLUD1 into intermediates that enter the tricarboxylic acid (TCA) cycle and generate α‐KG [7]. α‐KG functions as both a TCA cycle intermediate and a cofactor for multiple enzymes. It also regulates the epigenetic state of immune cells, thereby influencing cancer progression [8]. Compared with naïve macrophages, M2 macrophages exhibit greater dependence on glutamine metabolism. Targeting glutamine metabolism has therefore been proposed as a strategy to reprogram tumor‐associated macrophages (TAMs) from an M2 to an M1 phenotype, thereby enhancing antitumor immunity [9].

Recent studies have highlighted post‐translational modifications (PTMs), including lactylation, acetylation, and succinylation [10]. Among these, lysine lactylation is an emerging PTM that linkes cellular metabolic states to protein function. Through lactylation, lactate regulates diverse biological processes, including immune cell differentiation, tumor immune surveillance, and gene expression [11, 12]. Thus, tumor metabolic reprogramming and PTMs are closely interconnected [13].

USP22 is a member of the ubiquitin‐specific protease family that exerts its functions through deubiquitination and transcriptional regulation. We previously demonstrated that USP22 promotes cell proliferation, migration, and invasion and contributes to epidermal growth factor–tyrosine kinase inhibitor (EGFR‐TKI) resistance by preventing EGFR degradation in EGFR‐mutant lung adenocarcinoma cells [14]. Similarly, USP22 increases extracellular vesicles secretion and regulates the cell motility of lung adenocarcinoma [15].

The role of USP22 in tumor metabolic reprogramming has received increasing attention. USP22 modulates glycolysis and fatty acid metabolism, thereby supplying energy and biosynthetic precursors to tumor cells [16, 17]. In p53‐deficient colorectal cancer cells, reactive oxygen species (ROS)‐mediated upregulation of USP22 leads to deubiquitination and stabilization of FASN, promoting lipid synthesis and tumor growth [18]. In breast cancer, USP22 enhances autophagy (ATG)‐mediated lipolysis, accelerating catecholamine‐induced tumor progression and pulmonary metastasis [19]. USP22 also promotes metabolic reprogramming by regulating glycolytic transcription factors, enabling tumor cell adaptation to fluctuations in nutrient and oxygen availability within the tumor microenvironment [20]. However, despite substantial evidence linking USP22 to cancer metabolism, the molecular mechanisms by which it regulates glutamine metabolism remain unclear. In this study, we aimed to identify USP22 as a key regulator of glutamine metabolism and to evaluate its potential as a therapeutic target in TNBC.

## 2 Methods

### 2.1 Human TNBC Samples

Tissues from patients with TNBC were obtained from Harbin Medical University Cancer Hospital. All procedures were conducted with the approval of the Harbin Medical University Ethics Committee after written informed consent was collected from all patients.

### 2.2 Cell Culture and Transfection

All cell lines were maintained at Harbin Medical University Cancer Institute. BT549 and THP‐1 cells were cultured in RPMI 1640 medium (Gibco); MDA‐MB‐231, BT20, HEK‐293T, T47D, and SKBR3 cells were grown in DMEM (Gibco). All media contained 10% fetal bovine serum (FBS) and were maintained at 37°C with 5% CO_2_. Cell line authenticity was verified by short tandem repeat (STR) profiling. Lentiviral vectors were obtained from GeneChem, while SLC25A5 vectors were sourced from Hycyte. Scrambled lentiviral vectors served as negative controls. Transductions utilized polybrene followed by puromycin selection to establish stable lines. siRNA oligonucleotides and plasmids were sourced from RiboBio and GeneChem, respectively. All transfections were carried out using jetPRIME reagent according to the manufacturer's instructions. All siRNA and shRNA targeting sequences are provided in Table S1.

### 2.3 Animal Models

Animal experiments were performed with the approval of the Ethics Committee of the Institutional Animal Care and Use Committee of Harbin Medical University (No. YD2024‐12). Female BALB/c nude mice (4–5 weeks) were obtained from Vital River Laboratory.

For subcutaneous xenografts, 5×10^5^ cells in 100 µL PBS were implanted into the flanks. Tumor volume was measured weekly. For metastasis studies, 1×10^5^ 4T1‐Luc cells were intravenously injected via the tail vein. Metastasis was monitored every 3 days by bioluminescence imaging after intraperitoneal injection of 150 mg/kg luciferin (15 min before imaging) under anesthesia. Mice were sacrificed 4 weeks post‐injection for lung harvest and metastatic nodule analysis.

To examine the effect of K134‐pe or K134R‐pe on TNBC tumor growth, the mice were subcutaneously injected with 4T1 cells (5×10^5^ cells in 100 µL of PBS). After 7 days, the mice were randomized into three groups: a vehicle control group (saline), K134‐pe group (5 mg/kg), and K134R‐pe group (5 mg/kg). K134‐pe or K134R‐pe were intraperitoneally injected once a day.

The effect of K134‐pe combined with CB‐839 on TNBC tumor growth was examined. Seven days after tumor inoculation, the mice were randomly divided into four groups: a vehicle control group (saline), K134‐pe (5 mg/kg) group, CB‐839 (200 mg/kg) group, and K134‐pe (5 mg/kg) + CB‐839 (200 mg/kg) group. K134‐pe was intraperitoneally injected once a day, and CB‐839 was intraperitoneally injected twice a week. Tumor volume was measured as previously described.

### 2.4 RNA Isolation and qRT‐PCR

Total RNA was isolated using TRIzol reagent with chloroform phase separation, isopropanol precipitation, and ethanol washing. RNA quantity/quality was assessed by spectrophotometry. For qRT‐PCR, 1 µg RNA was reverse‐transcribed using a Transcriptor First Strand cDNA Synthesis Kit (Seven, Beijing, China). Real‐time PCR used cDNA, SYBR Green master mix, and gene‐specific primers under cycling conditions: 95°C (15 s); 40 cycles of 95°C (15 s). Melt curve analysis confirmed specificity. Gene expression was normalized to β‐actin using the ΔΔCt method. Table S2 presents the primers.

### 2.5 Metabolomics

Metabolites were extracted using ice‐cold methanol/acetonitrile/water (2:2:1, v/v/v) followed by centrifugation, with chromatographic separation performed on a ThermoFisher Ultimate 3000 ultrahigh‐performance liquid chromatography (UHPLC) system equipped with a Waters ACQUITY UPLC BEH Amide column using mobile phase A (90% acetonitrile containing 15 mM ammonium acetate and 0.2% ammonium hydroxide) and B (ultrapure water with identical additives) at a 0.30 mL/min flow rate. The following linear gradient elution procedure was used: 0–0.5 min: 5% B; 8 min: 30% B; 9 min: 50% B; 10.5 min: 50% B; 10.6 min: 5% B. The cells were maintained for 13 min. The eluates were separately analyzed on a Q Exactive HF‐X mass spectrometer in negative H‐ESI mode with spray voltage 4000 V, capillary/probe temperature 320°C, sheath gas 35 arb, auxiliary gas 10 arb, S‐Lens RF 50 arb, full scan range 70–1050 m/z at 70,000 FWHM (AGC 3e^6^), and top‐8 data‐dependent acquisition at 15/30/45 eV HCD with 17,500 FWHM (AGC 1e^5^), with data acquired via Xcalibur 4.1 and processed using TraceFinder 4.1.

### 2.6 Steady‐state Fractional Enrichment Analysis

MDA‐MB‐231 shCtrl and shUSP22 cells were cultured with glutamine‐free DMEM media and supplemented with 4 mM U‐13C glutamine (Cambridge Isotope Laboratories; CLM‐1822‐H), glucose, and 10% FBS for 6 h of incubation. Metabolic changes were then determined by UHPLC‐HRMS. Steady‐state fractional enrichment experiments were performed by Shanghai Profleader Biotechnology Co., LTD.

### 2.7 ECAR Measurement

Cells were seeded into Seahorse XF 96‐well plates at an appropriate density and cultured overnight at 37°C with 5% CO_2_. One day before the assay, the XF sensor cartridge was hydrated overnight at 37°C in a non‐CO_2_ incubator. On the day of the assay, culture medium was replaced with Seahorse XF base medium supplemented with 2 mm glutamine, and cells were incubated at 37°C without CO_2_ for 1 h. Glucose (10 mm), oligomycin (1 µm), and 2‐DG (50 mm) were loaded into the injection ports. After calibration, the assay was run on a Seahorse XF Pro analyzer using the Glycolysis Stress Test protocol, and ECAR was recorded in real time. At the end of the assay, total protein content was measured by BCA assay for normalization.

### 2.8 Detection of Intracellular Contents

Glutamate content, ATP level, α‐KG content, lactate content, and GLS enzyme activity were measured using their respective kits (BC1585, Solarbio; S0026, Beyotime; S0326, Beyotime; S0208, Beyotime; KTB3043, Abbkine, Wuhan, China). In brief, 5×10^6^ cells were collected, lysed with the corresponding lysates, and centrifuged for 30 min at 4°C. The supernatant was collected and added to the reaction solution, which was preincubated at 37°C. Optical density values were measured using a microplate reader.

### 2.9 Glutamine Uptake Assay

Glutamine levels in culture medium were measured using a glutamine assay kit (BC1455, Solarbio) per the manufacturer's instructions. Cells were seeded in 6‐well plates at 1×10^5^ cells/mL in fresh complete medium. Initial glutamine concentration (T0) was determined from fresh medium aliquots. After 24 h incubation, supernatants (T24) were collected. Cellular glutamine uptake was calculated as the difference between the T0 and T24 concentrations.

### 2.10 Macrophage Polarization

THP‐1 cells were differentiated into macrophages using 100 ng/mL PMA (MedChemExpress) for 24 h, then polarized to the M2 phenotype with 20 ng/mL IL‐4 (MedChemExpress) for 24 h.

### 2.11 Coculture Model

For conditioned medium (CM) preparation, cells were incubated in fresh serum‐free medium for 24 h. Supernatants were centrifuged (1000 ×g, 10 min, 4°C), filtered through 0.22 µm membranes, and stored as CM. Macrophages were cocultured with CM for 24 h, then harvested for qRT‐PCR and western blotting analysis.

### 2.12 Synergy Determination

BT20 and BT549 cells were seeded in 96‐well plates at 5000 cells per well. Cytotoxicity was assessed for single inhibitors (K134‐pe or CB‐839) or their combination at specified concentrations. Concentration gradients for K134‐pe and CB‐839 were established based on their respective IC50 value. After 72 h, viability was assessed via CCK‐8 assay. Synergy scores were computed using SynergyFinder (response surface model; Loewe method), with Loewe synergy >0 indicating synergy and >10 denoting strong synergy. Combinatorial effects were visualized through synergy heatmaps and 3D response landscapes.

### 2.13 Immunofluorescence (IF)

TNBC cells were plated on culture slides overnight. The cells or tissue samples were fixed in 4% formaldehyde (10 min), permeabilized with 0.1% Triton X‐100 (10 min), and blocked in 5% BSA (30 min). Primary antibodies were incubated overnight at 4°C. After incubation with fluorescent secondary antibodies (1 h, room temperature), nuclei were counterstained with DAPI. Samples were imaged using a Zeiss confocal microscope, with fluorescence intensity and colocalization analyzed.

### 2.14 Immunohistochemistry (IHC) and Hematoxylin and Eosin (HE) Staining

Tissue samples were paraffin‐embedded, baked at 65°C for 2 h, dewaxed in xylene, and hydrated through graded ethanol. Antigen retrieval was performed in preheated ethylenediaminetetraacetic acid (EDTA) buffer (pH 9.0, 98°C, 20 min). After cooling, endogenous peroxidase was blocked with 3% H_2_O_2_ (15 min), followed by PBS rinses and blocking with 5% goat serum (1 h, room temperature). Primary antibodies in blocking buffer were incubated overnight at 4°C. After PBS‐T washes, horseradish peroxidase (HRP)‐conjugated secondary antibodies were applied (1 h, room temperature). Signals were developed with DAB substrate (brown precipitate endpoint), counterstained with hematoxylin, dehydrated through graded alcohols, cleared in xylene, and mounted. Whole‐slide digital scans were semi‐quantitatively analyzed using the H‐score: staining intensity grade (0–3) × percentage of positive cells. Parallel HE staining enabled morphological assessment.

### 2.15 Cell Proliferation Assay

For CCK‐8, cells (8000/well in 96‐well plates) were incubated overnight, treated with interventions, then exposed to 1:10 diluted CCK‐8 reagent (UElandy Inc, Suzhou, China; C6005L) for 2 h (37°C, 5% CO_2_, dark); absorbance was measured at 450 nm. EdU proliferation was evaluated per the manufacturer's protocol (Beyotime). Colony formation assays used 600–1000 cells/well in 6‐well plates cultured for 2 weeks (37°C, 5% CO_2_), followed by paraformaldehyde fixation, crystal violet staining, and colony counting.

### 2.16 Transwell and Wound Healing Assays

Cell migration/invasion was assessed via Transwell and wound healing assays. For the Transwell assay, 5×10^4^ cells in 200 µL serum‐free medium were seeded in uncoated (migration) or Matrigel‐coated (invasion) chambers (8 µm pores) with 500 µL 10% FBS medium in lower chambers. After 24–48 h of incubation, migrated/invaded cells were fixed (4% PFA), crystal violet‐stained (0.5%), and microscopically quantified. For wound healing, confluent monolayers (2×10^6^ cells/well in 6‐well plates) were scratched with 10 µL pipette tips, cultured in serum‐free medium for 48 h, and imaged at 0/48 h. Wound closure rates were calculated from scratch width measurements using ImageJ.

### 2.17 Western Blotting

Western blotting analyses were performed as previously described [15] using USP22 (Abcam, ab195289; Proteintech, 55110‐1‐AP); Ki67 (Affinity Biosciences, AF0198); SLC25A5 (Santa Cruz, sc‐518109; Abclonal, A23411); CD206 (Proteintech, 18704‐1‐AP); Tip60 (Proteintech, 10827‐1‐AP); GLS (Abways, CY5719); SLC1A5 (Abclonal, A23156); GPR91 (Abways, AY4281); Pan‐Kla (PTM BIO, PTM‐1401RM); Flag (Abmart, M20008); Ubiquitin (Abcam, ab134853); His (Proteintech, 66005‐1‐Ig); PCBP4 (CUSABIO, CSB‐PA440349); PCAF (zen‐biosciencce, R382354); GCN5 (Biodragon, BD‐PT1875); P300 (Biodragon, BD‐PT5692); and TOMM20 (Abways, CY5527) antibodies.

### 2.18 Co‐Immunoprecipitation (Co‐IP) Assay and Mass Spectrometry (MS)

Protein interactions were analyzed by Co‐IP. Antibody‐bead complexes were formed by incubating primary antibodies with Protein A/G magnetic beads (4°C), then incubating overnight (4°C) with cell lysates (P70100, New Cell & Molecular Biotech) to capture target complexes. Beads were washed with precooled lysis buffer, and bound complexes were eluted for western blot analysis.

Co‐IP was performed using USP22‐specific antibodies versus an IgG control. Bound proteins were separated by SDS‐PAGE, target bands were digested in‐gel, and peptides were analyzed by LC‐MS/MS. USP22‐interacting candidates were identified via a UniProt database search using acquired peptide sequences.

### 2.19 GST Pulldown Assay

GST‐USP22 or the GST tag protein alone was purified from *E. coli* BL21 (Invitrogen) using GST agarose beads. Next, GST‐USP22 (the experimental group) or GST alone (the negative control group), both immobilized on GST agarose beads, were co‐incubated with His‐SLC25A5 protein, which was purified from HEK‐293T cells overnight at 4°C. Following incubation, the beads were washed three times with PBS buffer to remove unbound proteins. Finally, the bound complexes were analyzed by western blotting to verify the specific interaction between His‐SLC25A5 and GST‐USP22, as opposed to the GST control group.

### 2.20 Ubiquitination Assay

Endogenous SLC25A5 protein was enriched by IP with anti‐SLC25A5 antibody. Endogenous SLC25A5 ubiquitination level was assessed using western blotting with an anti‐ubiquitin antibody. A ubiquitinated plasmid was used to overexpress ubiquitin. After treating the cells with the proteasome inhibitor MG132 for 6 h and lysing, IP was performed using an anti‐SLC25A5 antibody. Ubiquitination levels were then analyzed by western blotting with the anti‐ubiquitin antibody.

### 2.21 In Vitro Lactylation Assay

Recombinant USP22 was incubated with recombinant Tip60 in lactylation buffer containing lactyl‐CoA. Reaction products were analyzed by Western blot using an anti‐USP22‐K134la antibody.

### 2.22 Proximity Ligation Assay (PLA)

Cells were fixed, permeabilized, and blocked to minimize background noise. Then, two primary antibodies produced in different host species were used to label target proteins. Species–specific PLA probes were added using a Duolink in situ PLA kit (Sigma–Aldrich, DUO92101) following the manufacturer's instructions. PLA signals were visualized using a confocal microscope (Zeiss).

### 2.23 Peptide Synthesis

All peptides were synthesized by QYAOBIO (ChinaPeptides Co., Ltd.) and purified by high‐performance liquid chromatography to ≥98% purity. All peptides used in the in vivo experiments were synthesized from D‐configuration amino acids.

### 2.24 Molecular Docking

The structures of the key target proteins were obtained from the Protein Data Bank database (https://www.rcsb.org/). Pymol software was used to dehydrate the proteins and extract the target protein structure. The protein was obtained from the GRAMM online server (https://gramm.compbio.ku.edu/), and LigPlot+ software was used to analyze the chemical bonds in the results. Pymol software was then used to visualize the results.

### 2.25 Bioinformatics Analysis

The survival data for patients with TNBC were downloaded from GEPIA (http://gepia2.cancer‐pku.cn) and analyzed using Kaplan–Meier analysis and the log‐rank test. The correlation between USP22 and SLC25A5 expression in TNBC was determined through GEPIA and TIMER (http://timer.cistrome.org). GSE45827 was used to evaluate the degree of glutamine metabolism in different breast cancer subtypes. GSE65194, GSE76275, and GSE103091 were used to assess the expression of USP22 and SLC25A5 in TNBC cells. Based on 73 identified glutamine metabolism‐related genes, the degree of glutamine metabolism was calculated using the Gene Set Variation Analysis (GSVA) method, and the 73 glutamine metabolism‐related genes were extracted from the Molecular Signature Database (MSigDB, http://www.gsea msigdb.org/gsea/msigdb/index.jsp).

### 2.26 Statistical Analysis

All experiments were independently repeated thrice. The unpaired two‐tailed Student's t‐test was used to compare the two groups, and one‐way or two‐way analysis of variance (ANOVA) was used to compare multiple groups using GraphPad Prism software. Data were expressed as mean ± standard deviation (SD), and statistical significance was defined as *p* < 0.05.

## 3 Results

### 3.1 USP22 Increases Glutamine Uptake and Promotes Tumor Progression

Bioinformatics analysis of Gene Expression Omnibus (GEO) data revealed significant upregulation of genes related to glutamine metabolism in breast tumor tissues (Figure S1A), particularly in TNBC (Figure S1B), where high activity correlates with a poor prognosis (Figure S1C). TNBC cells depend on glutamine metabolism for growth, migration, and invasion (Figure S1D–I), highlighting the critical role of glutamine metabolism in TNBC pathogenesis.

To investigate the role of USP22, we established stable knockdown and overexpression TNBC cell lines (Figure S2A,B). USP22 knockdown inhibited proliferation, migration, and invasion in vitro, whereas overexpression promoted these phenotypes (Figures S2C–E and S3A,B). Consistently, USP22 knockdown attenuated lung metastasis in vivo (Figure S3C–F).

Proteomic and metabolic profiling of USP22‐knockdown MDA‐MB‐231 cells revealed widespread changes in amino acid metabolism and tumor‐related pathways (Figure 1A–E). ^1^ ^3^C‐glutamine tracing showed that USP22 knockdown reduced glutamine uptake and catabolism, with decreased levels of α‐KG and other TCA cycle intermediates (Figure 1F,G). Further validation confirmed that USP22 knockdown increased supernatant glutamine but decreased intracellular glutamate and α‐KG, and suppressed the enzymatic activities of GLS, GLUD1, and GOT; overexpression had the opposite effects (Figure 1H–K and Figure S4A–G). Seahorse assays further demonstrated that USP22 regulates glycolysis, and USP22 depletion reduced cellular ATP and lactate (Figure 1L–O and Figure S4H–J).

**FIGURE 1:**
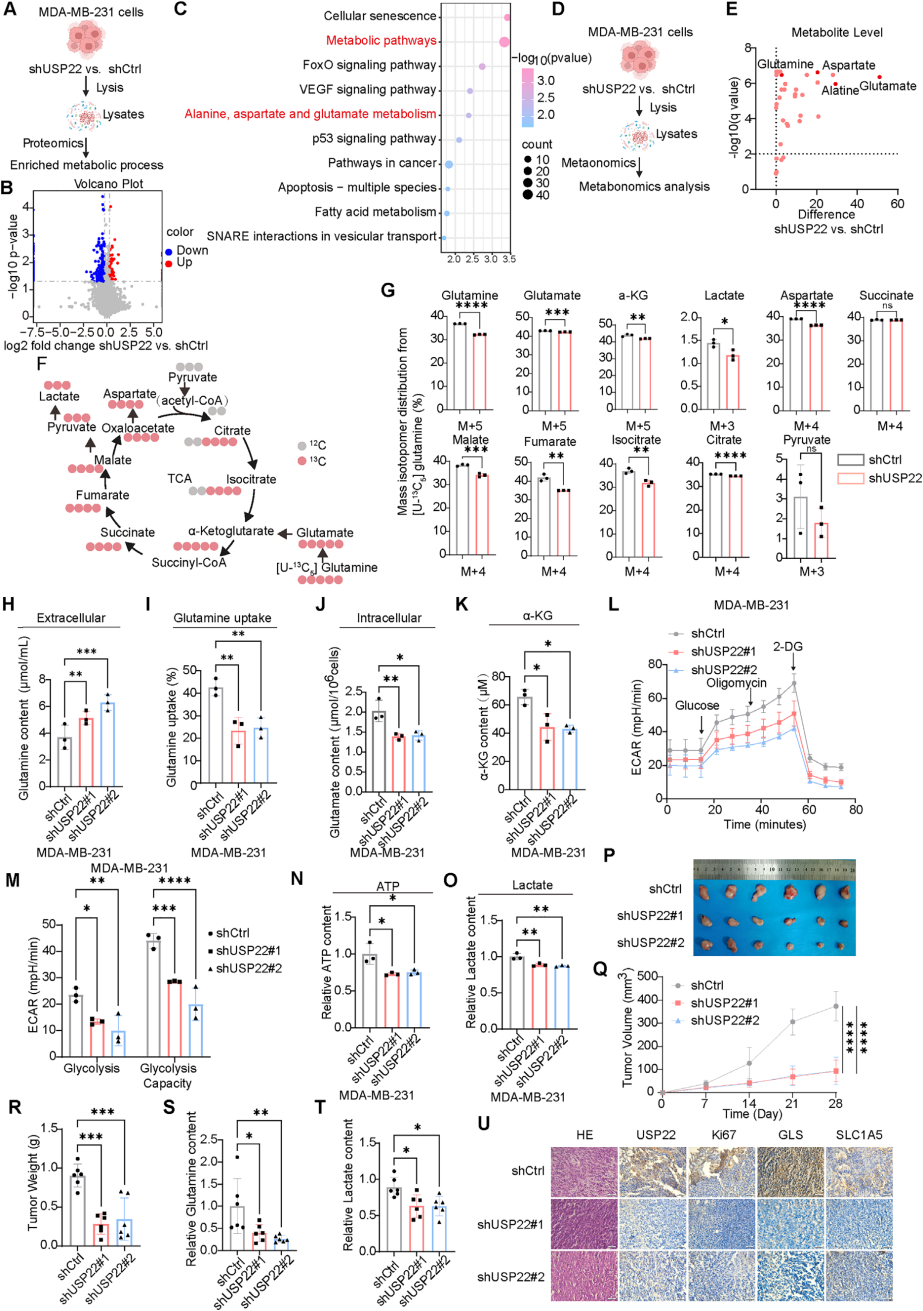
USP22 regulates glutamine metabolism reprogramming in TNBC. (A) Proteomic analysis of USP22 knockdown cells and MDA‐MB‐231 control (ctrl) cells. (B) DEPs in the USP22 knockdown and control MDA‐MB‐231 cells. (C) DEPs between the shUSP22 and shCtrl groups. (D) USP22 knockdown cells and MDA‐MB‐231 ctrl cells. (E) Differential metabolites of shUSP22 and shCtrl group in MDA‐MB‐231 cells. (F) Glutamine metabolism pathway and TCA cycle intermediates. ^13^C‐glutamine [U‐^13^C_5_]‐labeled carbons (pink). (G) The abundance of glutamine‐derived and total metabolites in shCtrl and shUSP22 MDA‐MB‐231 cells. MDA‐MB‐231 cells were pre‐cultured in a glutamine‐free medium for 20 h. Then, the cells were incubated in a labeling medium containing 4 mM of [U‐^13^C_5_] glutamine for 6 h. (H) Glutamine concentration in cell culture supernatants. (I) Glutamine uptake in shCtrl and shUSP22 MDA‐MB‐231 cells. (J,K) Glutamate content (J) and α‐KG content (K) of MDA‐MB‐231 cells with or without USP22 knockdown. (L,M) Seahorse ECAR analysis to evaluate glycolytic phenotypes upon USP22 knockdown in MDA‐MB‐231 cells. (N,O) ATP production and lactate levels in shCtrl and shUSP22 MDA‐MB‐231 cells. (P–R) Tumor images (P), volumes (Q), and weights (R) of 4T1‐derived xenograft tumors (n = 6). (S) Glutamine content in tumor tissues. (T) Lactate content in tumor tissues (per 10 mg tissue). (U) IHC staining for USP22, Ki67, GLS, and SLC1A5 in subcutaneous tumors. Scale bars, 50 µm. Statistical significance was determined using an unpaired two‐tailed Student's t‐test for G; one‐way ANOVA test for H, I, J, K, N, O, R, S, and T; and two‐way ANOVA test for M and Q. Data are expressed as means ± SD. ^*^ *p* < 0.05; ^**^ *p* < 0.01; ^***^ *p* < 0.001; ^****^ *p* < 0.0001; ns, no statistical significance.

In subcutaneous xenograft models, USP22 knockdown impaired tumor growth and reduced intra‐tumoral glutamine and lactate (Figure 1P–T). IHC confirmed lower USP22, Ki67, SLC1A5, and GLS expression in knockdown tumors (Figure 1U). Together, these findings demonstrate that USP22 drives TNBC proliferation and invasion by upregulating glutamine metabolism.

### 3.2 Glutamine Drives Lactate‐Induced USP22 K134 Lactylation

Glutamine upregulated USP22 protein in a dose‐dependent manner without altering its mRNA levels, indicating a posttranslational regulatory mechanism (Figure 2A,B). Consistent with this, glutamine supplementation promoted USP22 lactylation and enhanced its colocalization with Pan‐Kla (Figure 2C,D). Clinically, elevated Pan‐Kla and USP22 correlated with high‐risk TNBC and were significantly increased in tumor tissues compared to adjacent normal tissues (Figure S5A). Moreover, IHC staining revealed a positive correlation between Pan‐Kla and USP22 expression in these tissues, whereas USP22 knockdown reduced Pan‐Kla in subcutaneous tumors (Figure S5B–F). Thus, glutamine promotes tumorigenesis by driving USP22 lactylation.

**FIGURE 2:**
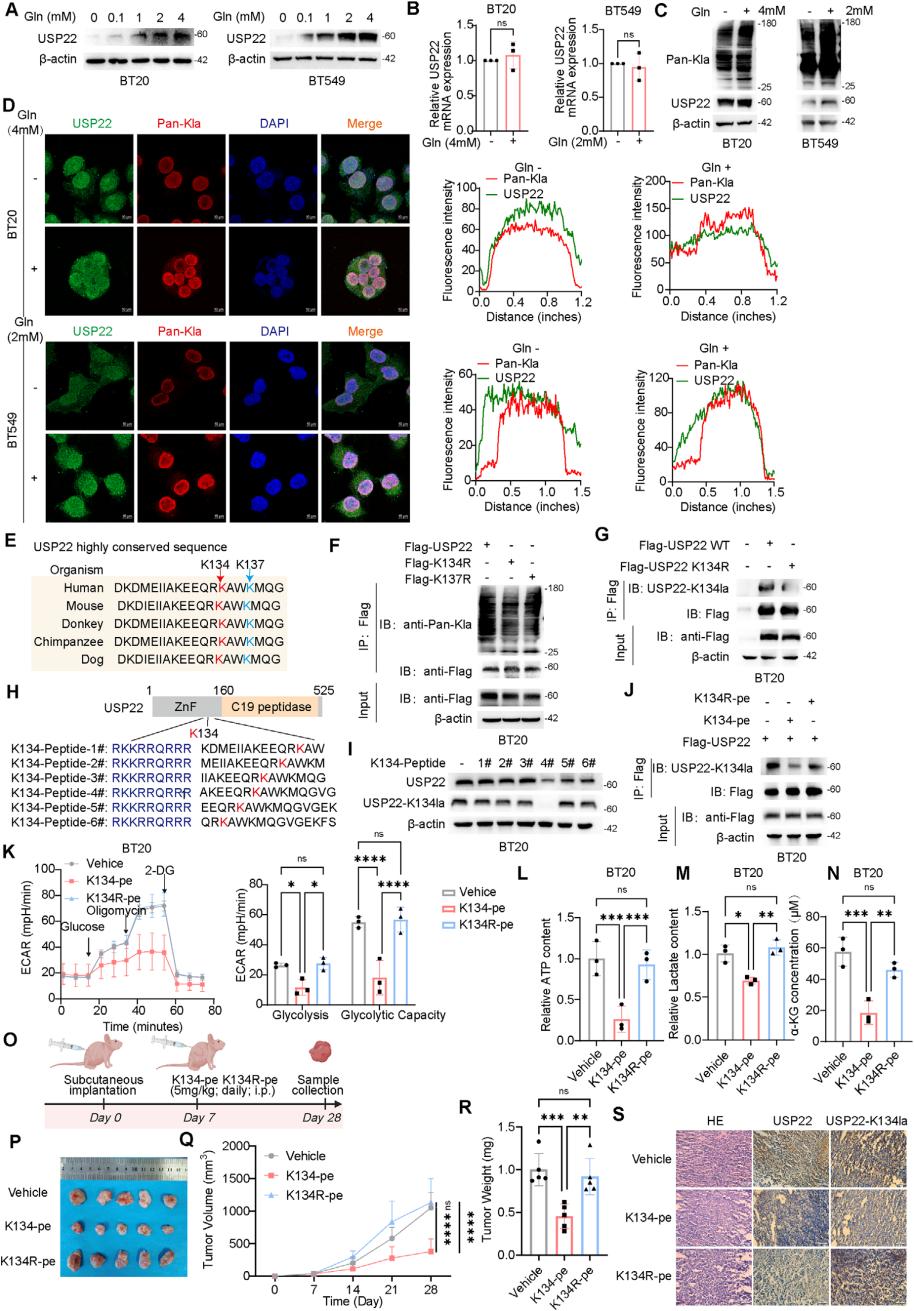
Glutamine increases USP22 expression by inducing lactylation. (A) The expression levels of the USP22 protein in BT20 and BT549 cells analyzed after treatment with different concentrations of glutamine. (B) Expression of mRNA of USP22. (C) Effect of glutamine on USP22 lactation. (D) Colocalization of USP22 and Pan‐Kla in BT20 and BT549 cells, with and without glutamine. Scale bars, 10 µm. (E) Sequence alignment of the conserved regions containing K134 and K137 in USP22 homologues from different species. (F,G) Lactylation site of USP22. (H) Amino acid sequences of the designed peptide. Blue: cell‐penetrating peptide. Red: site for USP22 lactylation. (I,J) Site for USP22 lactylation treated with K134‐peptide (10 µm) for 24 h. (K) Seahorse ECAR analysis to evaluate glycolytic phenotypes. (L–N) ATP level (L), lactate content (M), and α‐KG content (N) of BT20 cells treated with K134‐peptide or K134R‐peptide (10 µm) for 24 h. (O) Schematic representation of subcutaneous xenograft tumor model. (P–R) Tumor images (P), volumes (Q), and weights (R) of 4T1‐derived xenograft tumors (*n* = 5). (S) IHC staining for USP22 and USP22‐K134la in subcutaneous tumors. Scale bars, 50 µm. Statistical significance was determined using an unpaired two‐tailed Student's t‐test for B; one‐way ANOVA test for K, L, M, N, and R; and two‐way ANOVA test for Q. Data are expressed as means ± SD. ^*^ *p* < 0.05; ^**^ *p* < 0.01; ^***^ *p* < 0.001; ^****^ *p* < 0.0001; ns, no statistical significance.

To pinpoint the specific lactylation site, we identified two conserved candidates, K134 and K137 (Figure 2E). To further confirm the site of lactylation, transfecting BT20 cells with USP22 mutants (K134R, K137R) revealed that the K134 mutation drastically reduced lactylation levels, identifying K134 as the primary site (Figure 2F). To recognize USP22 lactylation at K134, an antibody (anti‐USP22 K134la) was prepared. The USP22 K134 mutant completely decreased the signal detected by this antibody (Figure 2G).

We designed six cell‐penetrating peptides based on the K134 flanking sequence (Figure 2H) [21]. Screening identified K134‐peptide‐4# (K134‐pe) as the most potent inhibitor of USP22 lactylation, whereas a scrambled control (K134R‐pe) had no effect (Figure 2I,J). Consequently, K134‐pe, but not K134R‐pe, disrupted glutamine metabolism by suppressing glycolytic capacity, lactate and ATP production, and reducing α‐KG (Figure 2K–N and Figure S6A–D). This confirms that K134‐pe inhibits glutamine metabolism by blocking USP22 K134 lactylation. Functionally, K134‐pe selectively inhibited TNBC proliferation, migration, and invasion (Figure S6E–H) and significantly suppressed tumor growth in vivo compared to K134R‐pe (Figure 2O–S). Therefore, targeting USP22 K134 lactylation with K134‐pe disrupts glutamine metabolism to exert antitumor effects, establishing USP22 K134 lactylation as a promising therapeutic target.

### 3.3 Tip60 Catalyzes USP22 K134 Lactylation

Glutamine‐derived lactate serves as the lactyl group donor for protein lactylation [22]. To identify the lactyltransferase mediating glutamine‐induced USP22 lactylation, we screened five major eukaryotic lactyltransferases [23]. Tip60 emerged as the key candidate, as only its depletion reduced USP22 levels (Figure 3A). We then performed molecular docking to confirm the interaction between USP22 and Tip60 (Figure 3B). Co‐IP assays showed that Tip60 interacted with both endogenous and exogenous USP22, and USP22 likewise interacted with Tip60 (Figure 3C,D). We observed localization of USP22 and Tip60 in cells using confocal fluorescence microscopy; the two proteins were colocalized within BT20 and BT549 cells (Figure 3E).

**FIGURE 3:**
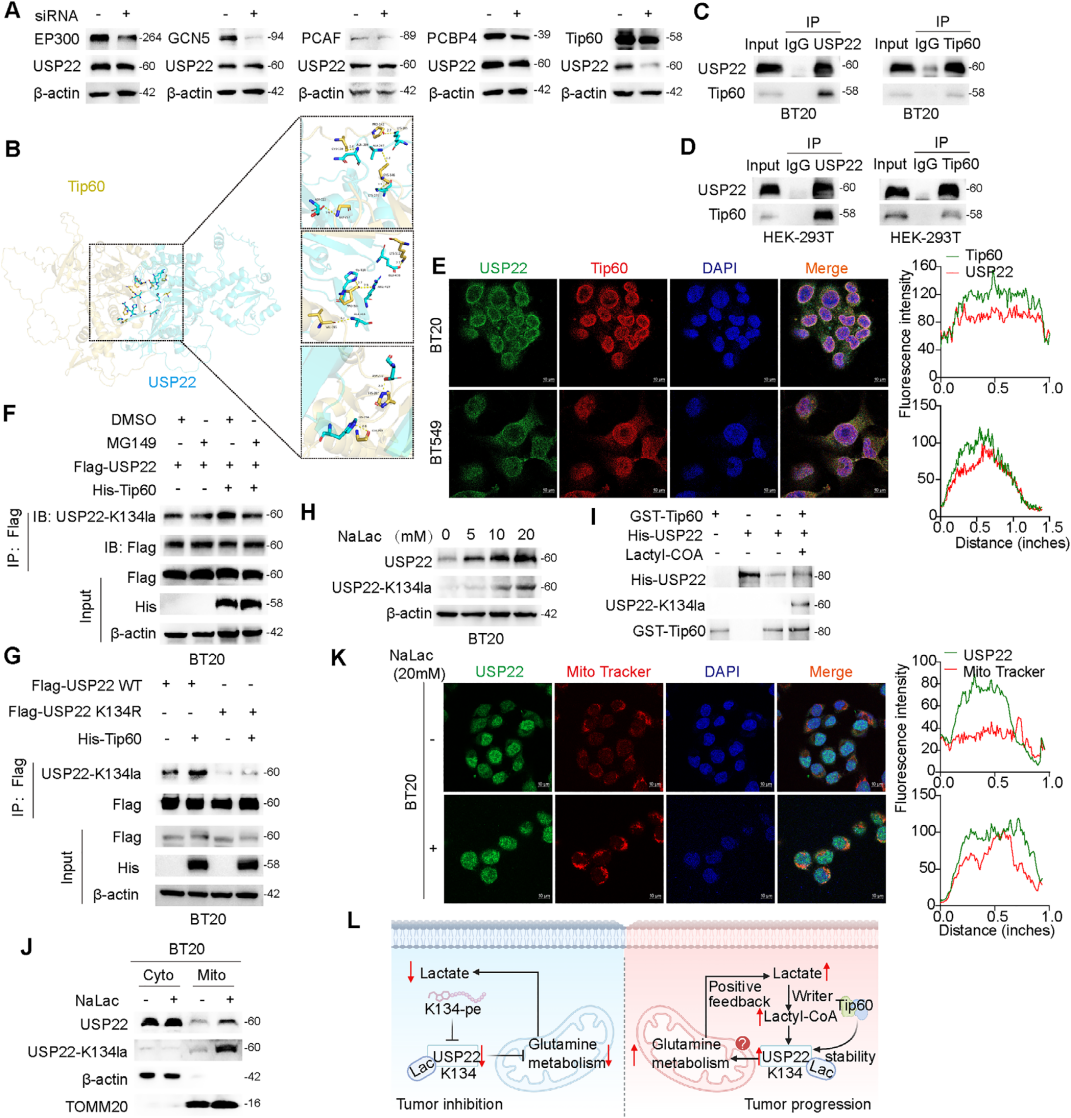
Tip60 regulates USP22 K134 lactylation. (A) BT20 cells transfected with control siRNA or siRNAs targeting EP300, GCN5, PCAF, PCBP4, or Tip60. (B) Interactions between USP22 and Tip60. (C) USP22 interacting with Tip60 in BT20 cells. (D) USP22 interacting with Tip60 in HEK‐293T cells. (E) Colocalization of USP22 and Tip60. Scale bars, 10 µm. (F) Flag‐USP22 WT and His‐Tip60 are co‐expressed in BT20 cells with or without MG149 (10 µm) for 24 h. The cell lysates were subjected to IP with anti‐Flag, followed by western blotting. (G) His‐Tip60 and Flag‐USP22 WT or Flag‐USP22 K134R mutants were co‐overexpressed in BT20 cells. The cell lysates were subjected to IP with anti‐Flag, followed by western blotting analysis. (H) USP22 K134 lactylation in BT20 cells treated with indicated concentrations of sodium lactate for 24 h. (I) In vitro lactylation assay showing lysine lactyltransferase activity of Tip60. (J) Comparison of the subcellular distribution of USP22 in the mitochondria and cytoplasm of BT20 cells after 24 h of culture under conditions of adequate or deficient sodium lactate. (K) Localization of USP22 and Mitotracker in BT20 cells under control conditions and in the absence of sodium lactate. Scale bars, 10 µm. (L) Tip60‐mediated USP22 lactylation promotes tumor glutamine metabolism reprogramming. Glutamine‐derived lactate regulates USP22 lactylation.

Functionally, Tip60 overexpression increased USP22 lactylation, whereas the Tip60 inhibitor MG149 reduced K134 lactylation (Figure 3F). Tip60 enhanced K134 lactylation of wild‐type USP22 but failed to modify the lactylation‐deficient K134R mutant (Figure 3G). Therefore, Tip60 is the primary lactyltransferase targeting USP22 K134 and promoting USP22 stability. We further found that K134 lactylation increased in a dose‐dependent manner upon sodium lactate stimulation (Figure 3H). In vitro lysine lactylation confirmed that Tip60 mediated USP22 K134 lactylation (Figure 3I). This lactate‐driven modification was predominantly mitochondrial, with USP22 expression and K134 lactylation preferentially enhanced in mitochondria and minimal cytoplasmic changes (Figure 3J). Consistent with this mitochondrial preference, sodium lactate stimulation increased USP22 colocalization with mitochondria (Figure 3K). Thus, glutamine‐derived lactate promotes Tip60‐mediated USP22 K134 lactylation, driving USP22 mitochondrial translocation and metabolic reprogramming (Figure 3L).

### 3.4 USP22 Interacts With SLC25A5

Glutamine can be synthesized intracellularly by GLUL or imported through transporters such as SLC1A5 and SLC38A1 [24], and mitochondria serve as central hubs for glutamine catabolism and anabolism [25]. The transport of metabolites across the mitochondrial inner membrane (IMM) is strictly regulated [26]. Since the IMM is impermeable to metabolites, their translocation relies on specific membrane‐bound carrier proteins. SLC25A proteins are the largest family of carrier proteins in mitochondria, primarily localized to the IMM [27]. In an unbiased mass spectrometry screen in MDA‐MB‐231 cells, SLC25A5 emerged as a prominent USP22‐associated protein relative to IgG control (Figure 4A,B). We therefore asked whether USP22 and SLC25A5 physically interact. Molecular docking predicted a potential interaction between the two proteins (Figure 4C). Co‐IP in BT20 and MDA‐MB‐231 cells supported this association (Figure 4D,E), and reciprocal GST/His pull‐down assays demonstrated direct binding (Figure 4F). Confocal imaging showed that USP22 and SLC25A5 colocalized mainly in the cytoplasm (Figure 4G). Proximity ligation assays produced distinct signals that were enhanced by USP22 overexpression, further supporting direct binding (Figure 4H). Furthermore, overexpression of USP22 enhanced the interaction between USP22 and SLC25A5 (Figure 4I). Glutamine supplementation increased USP22 levels in the cytoplasm and mitochondria, while SLC25A5 was confined to mitochondria (Figure 4J). We then treated isolated mitochondrial fractions with trypsin. Following glutamine supplementation, trypsin was unable to effectively hydrolyze the USP22 protein, suggesting that a portion of USP22 was associated with a trypsin‐protected mitochondrial compartment (Figure 4K). Consistently, glutamine enhanced USP22‐SLC25A5 colocalization (Figure 4L). Therefore, USP22 can bind to SLC25A5 and form a complex in cells.

**FIGURE 4:**
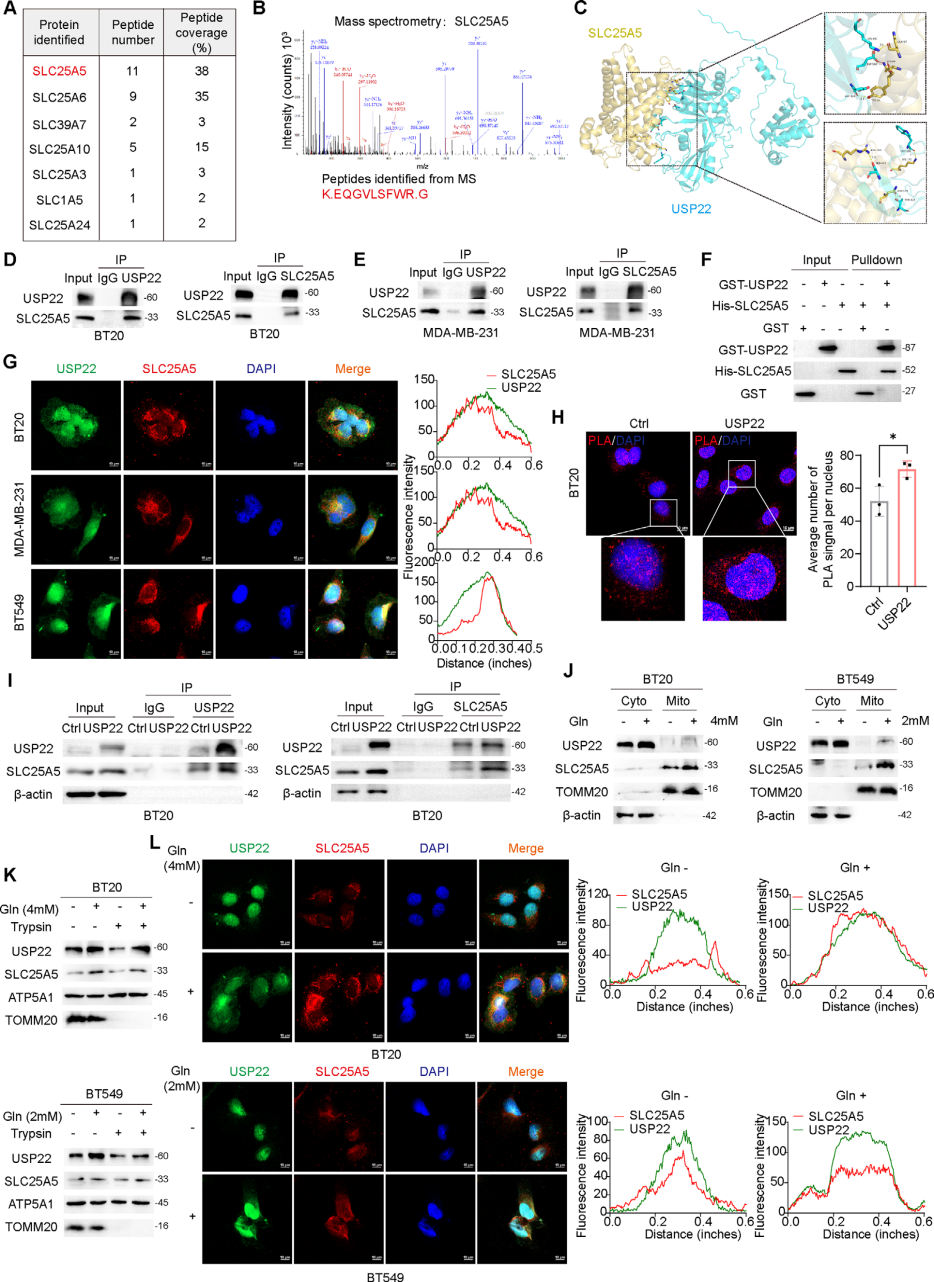
USP22 directly interacts with SLC25A5. (A) SLCs proteins. The protein of interest is highlighted in red. (B) SLC25A5‐specific peptide sequences. (C) USP22‐SLC25A5 interaction. (D,E) Confirmed USP22‐SLC25A5 interaction in BT20 and MDA‐MB‐231 cells. (F) GST pulldown assay: Purified His‐SLC25A5 was incubated with glutathione beads bound to GST or GST‐USP22. Proteins retained on the beads were analyzed. (G) Colocalization of USP22 and SLC25A5 in TNBC cells. Scale bars, 10 µm. (H) USP22‐SLC25A5 interaction. Scale bars, 10 µm. (I) BT20 cells transfected with or without USP22 overexpression plasmid were immunoprecipitated with anti‐USP22 or anti‐SLC25A5 antibody, and the interaction between USP22 and SLC25A5 was detected. (J) Subcellular distribution of USP22 in the mitochondrial and cytoplasmic fractions of BT20 cells cultured under glutamine‐sufficient or glutamine‐deprived conditions for 24 h was compared. (K) Mitochondrial fractions from cells under glutamine‐deprived or glutamine‐sufficient conditions. Mitochondria were treated with or without trypsin to lyse the outer mitochondrial membrane (OMM). USP22 levels were compared to markers of the inner mitochondrial membrane (IMM; ATP5A1) and OMM (TOMM20). (L) USP22‐SLC25A5 colocalization in TNBC cells under control or glutamine‐deprived conditions. Scale bars, 10 µm. Statistical significance was determined using an unpaired Student's t‐test for H. Data are expressed as means ± SD. ^*^ *p* < 0.05.

### 3.5 SLC25A5 Promotes TNBC Progression and Glutamine Metabolism

To investigate the role of SLC25A5 in TNBC, we established stable SLC25A5‐knockdown BT20 cells and SLC25A5‐overexpressing BT549 cells, and confirmed the efficiency of both models (Figure S7A,B).

Knockdown of SLC25A5 in BT20 cells significantly inhibited TNBC cell proliferation, migration, and invasion abilities. Conversely, overexpression of SLC25A5 in BT549 cells led to a marked enhancement of these malignant phenotypes (Figure S7C–G). Therefore, SLC25A5 serves as a key gene promoting TNBC progression. To determine whether SLC25A5 contributes to glutamine metabolism, we measured glutamine uptake, mitochondrial glutamate content, and intracellular α‐KG levels. SLC25A5 knockdown decreased glutamine uptake, mitochondrial glutamate content, and intracellular α‐KG levels, whereas SLC25A5 overexpression increased all these parameters (Figure S7H–J). These results indicate that SLC25A5 positively regulates glutamine uptake and helps maintain mitochondrial glutamate and α‐KG pools.

### 3.6 USP22 Stabilizes SLC25A5 Expression

USP22 knockdown decreased SLC25A5 protein levels, whereas its overexpression produced the opposite effect (Figure 5A). SLC25A5 mRNA levels were not significantly altered by USP22 knockdown or overexpression (Figure 5B), indicating that USP22 post‐translationally regulates SLC25A5. USP22 knockdown markedly shortened the SLC25A5 half‐life, while USP22 overexpression extended it (Figure 5C). The reduction in SLC25A5 protein induced by USP22 knockdown was reversed by the proteasome inhibitor MG132 (Figure 5D). Furthermore, USP22 knockdown elevated SLC25A5 ubiquitination in MDA‐MB‐231 and BT20 cells. Conversely, USP22 overexpression reduced ubiquitination in BT549 and BT20 cells (Figure 5E). Therefore, USP22 stabilizes SLC25A5 protein by inhibiting its ubiquitin‐mediated proteasomal degradation.

**FIGURE 5:**
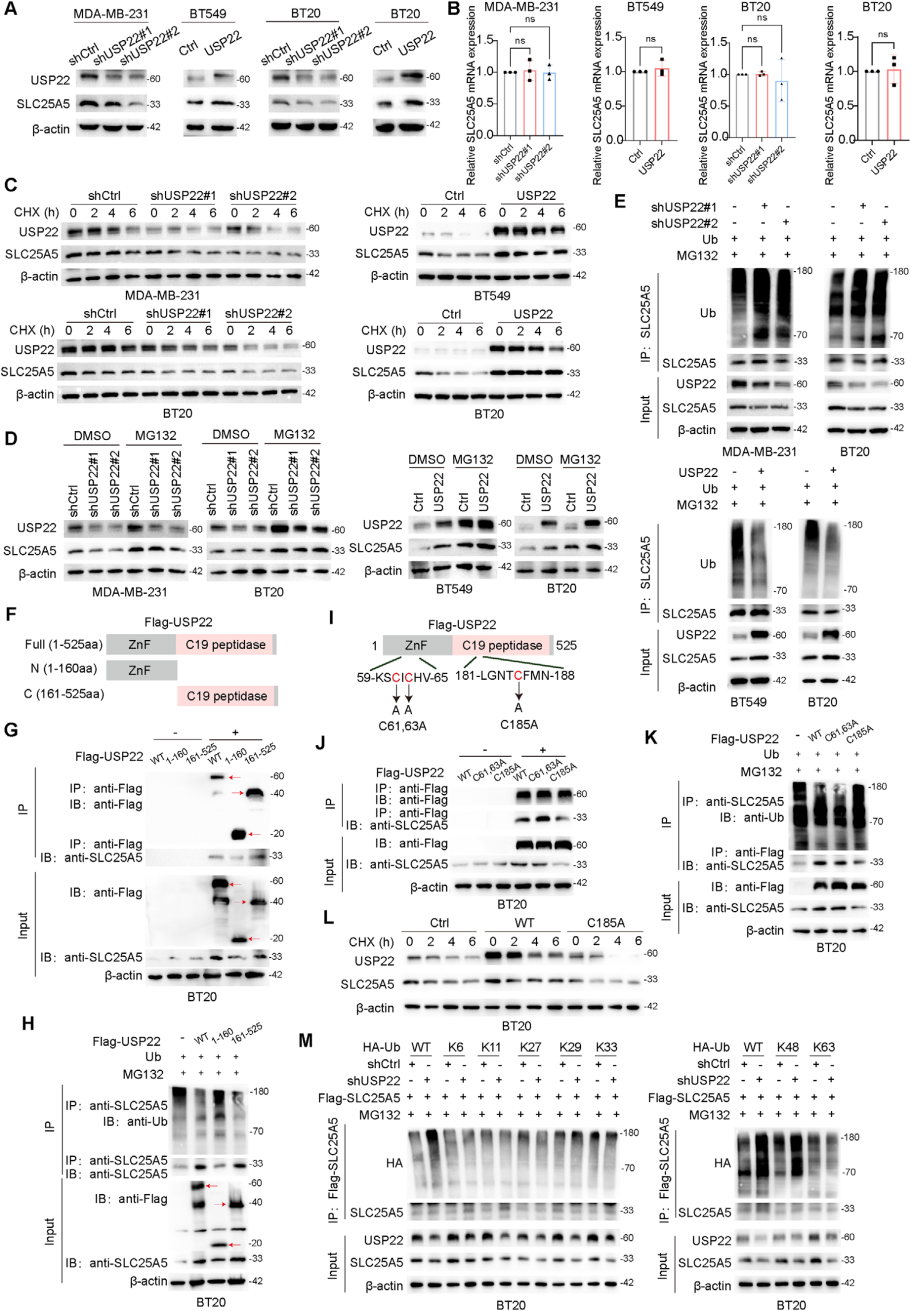
USP22 prevents SLC25A5 degradation through deubiquitination. (A) SLC25A5 protein expression in TNBC cells following USP22 knockdown or overexpression. (B) SLC25A5 mRNA expression in TNBC cells after USP22 knockdown or overexpression. (C) Western blot analysis of SLC25A5 protein degradation treated with CHX (200 µg/mL). (D) SLC25A5 stability following proteasomal inhibition with MG132 (10 µm, 6 h). (E) Effect of USP22 knockdown or overexpression on SLC25A5 ubiquitination levels. (F) Structures of USP22 and its truncated mutants. (G) Binding domains of USP22 and SLC25A5. (H) Detection of SLC25A5 ubiquitination. (I) USP22 and its catalytic point mutants. (J) Interaction analysis between SLC25A5 and USP22 or its point mutants. (K) USP22 wild‐type or point mutants are transfected to analyze SLC25A5 ubiquitination in BT20 cells. (L) SLC25A5 and USP22 protein levels detected in BT20 cells treated with CHX. (M) BT20 cells co‐transfected with HA‐Ub WT, or HA‐K6‐Ub (Lys6‐only), or HA‐K11‐Ub (Lys11‐only), or HA‐K27‐Ub (Lys27‐only), or HA‐K29‐Ub (Lys29‐only), or HA‐K33‐Ub (Lys33‐only), or HA‐K48‐Ub (Lys48‐only), or HA‐K63‐Ub (Lys63‐only) plasmids. The SLC25A5 ubiquitylation linkage was detected using IP analysis with anti‐HA antibody, followed by treatment with MG132 for 6 h. Statistical significance was determined using an unpaired Student's t‐test. Data are expressed as means ± SD. ns, no statistical significance.

To identify the USP22 region interacting with SLC25A5, we generated two USP22 truncated mutants (Figure 5F). Co‐IP revealed that the C19 domain bound strongly to SLC25A5, whereas the N‐terminal domain bound weakly (Figure 5G). Functionally, the C19 domain was essential for USP22‐mediated deubiquitination of SLC25A5 (Figure 5H). Next, we constructed USP22 point mutants (Figure 5I). Consistent with the truncation data, mutating the C19 domain impaired SLC25A5 binding, whereas mutating the zinc‐finger domain enhanced it (Figure 5J). Moreover, compared to wild‐type USP22, the C19 domain mutant (C185A) failed to remove ubiquitin chains from SLC25A5, resulting in sustained high ubiquitination levels (Figure 5K). Consequently, this mutant lost the ability to stabilize SLC25A5 protein following CHX treatment (Figure 5L). USP22 specifically cleaved K48‐linked polyubiquitin chains on SLC25A5, with minimal activity against chains linked via K6, K11, K27, K29, K33, or K63 (Figure 5M).

Tip60 co‐expression enhanced the effect of USP22 overexpression on the deubiquitination of SLC25A5 in BT20 cells (Figure S8A). Overexpression of USP22 K134R led to an increase in the ubiquitination level of SLC25A5 (Figure S8B). Co‐overexpression of Tip60 and USP22 enhanced SLC25A5 stability (Figure S8C). Similarly, the overexpression of the USP22 K134R mutant significantly decreased the stability of the SLC25A5 protein in BT20 cells (Figure S8D). Therefore, the C19 ubiquitin‐specific peptidase domain of USP22 primarily mediated its interaction with SLC25A5 and catalyzed the removal of K48‐linked polyubiquitin chains from SLC25A5. Tip60 was the key lactyltransferase of USP22 and enhanced the stability of both USP22 and SLC25A5 by targeting the USP22 K134 site.

### 3.7 USP22 Expression Correlates With SLC25A5 in Patients With TNBC

To assess the clinical relevance of the USP22‐SLC25A5 axis in TNBC, we examined patient tumor tissues (Figure S9A). Western blotting analysis revealed high expression of both USP22 and SLC25A5 in tumor tissues (Figure S9B). USP22, SLC25A5, and USP22 K134la were highly expressed in TNBC tissues (Figure S9C–F). Notably, USP22‐K134la was also positively correlated with both USP22 and SLC25A5 (Figure S9G–I). Analysis of the TIMER database also confirmed the significant positive correlation between USP22 and SLC25A5 (Figure S9J). GEPIA2 database results showed that patients with high expression of both USP22 and SLC25A5 had significantly shorter OS, suggesting that high expression of these genes was associated with poor prognosis (Figure S9K–M). In the high‐risk glutamine metabolism group, USP22 and SLC25A5 expression levels were significantly higher (Figure S9N–P). USP22 and SLC25A5 were independently associated with a poor prognosis in patients with TNBC (Figure S9Q,R). The prognosis was the worst when both were highly expressed (Figure S9S). The role of USP22 in TNBC is supported by these findings through the regulation of SLC25A5, and both are significantly associated with a poor prognosis in patients with TNBC.

### 3.8 SLC25A5 Is Required for USP22‐Mediated Glutamine Metabolism

USP22 overexpression significantly increased SLC25A5 mitochondrial localization (Figure 6A), while USP22 knockdown reduced it (Figure S10A). We performed proteomic analysis and showed that SLC25A5 overexpression specifically activated the nitrogen metabolism pathway (Figure 6B,C), a pathway in which GLS catalyzes the rate‐limiting hydrolysis of glutamine to glutamate and ammonia during glutaminolysis (Figure 6D). Functionally, USP22 knockdown inhibits TNBC cell migration and invasion, while its overexpression promotes these effects. Both phenotypes were reversible by modulating SLC25A5 expression (Figure S10B,C). SLC25A5 knockdown suppressed USP22‐driven glutamine metabolism and reduced SLC1A5 and GLS expression (Figure 6E). Conversely, SLC25A5 overexpression reversed the effects of USP22 knockdown in BT20 cells (Figure S10D). SLC25A5 knockdown blocked the increase in α‐KG, ATP, and lactate levels induced by overexpressing USP22 (Figure 6F–H). Conversely, SLC25A5 overexpression rescued their decrease upon USP22 knockdown (Figure S10E–G). USP22 overexpression significantly promoted subcutaneous tumor growth, an effect reversed by SLC25A5 knockdown in vivo (Figure 6I–L). IHC analysis confirmed downregulation of Ki67, SLC1A5, and GLS after SLC25A5 knockdown (Figure 6M). USP22 overexpression increased metastatic nodules, which were reversed by SLC25A5 knockdown (Figure 6N–Q). Therefore, SLC25A5 interacts with USP22 to promote tumor progression in TNBC.

**FIGURE 6:**
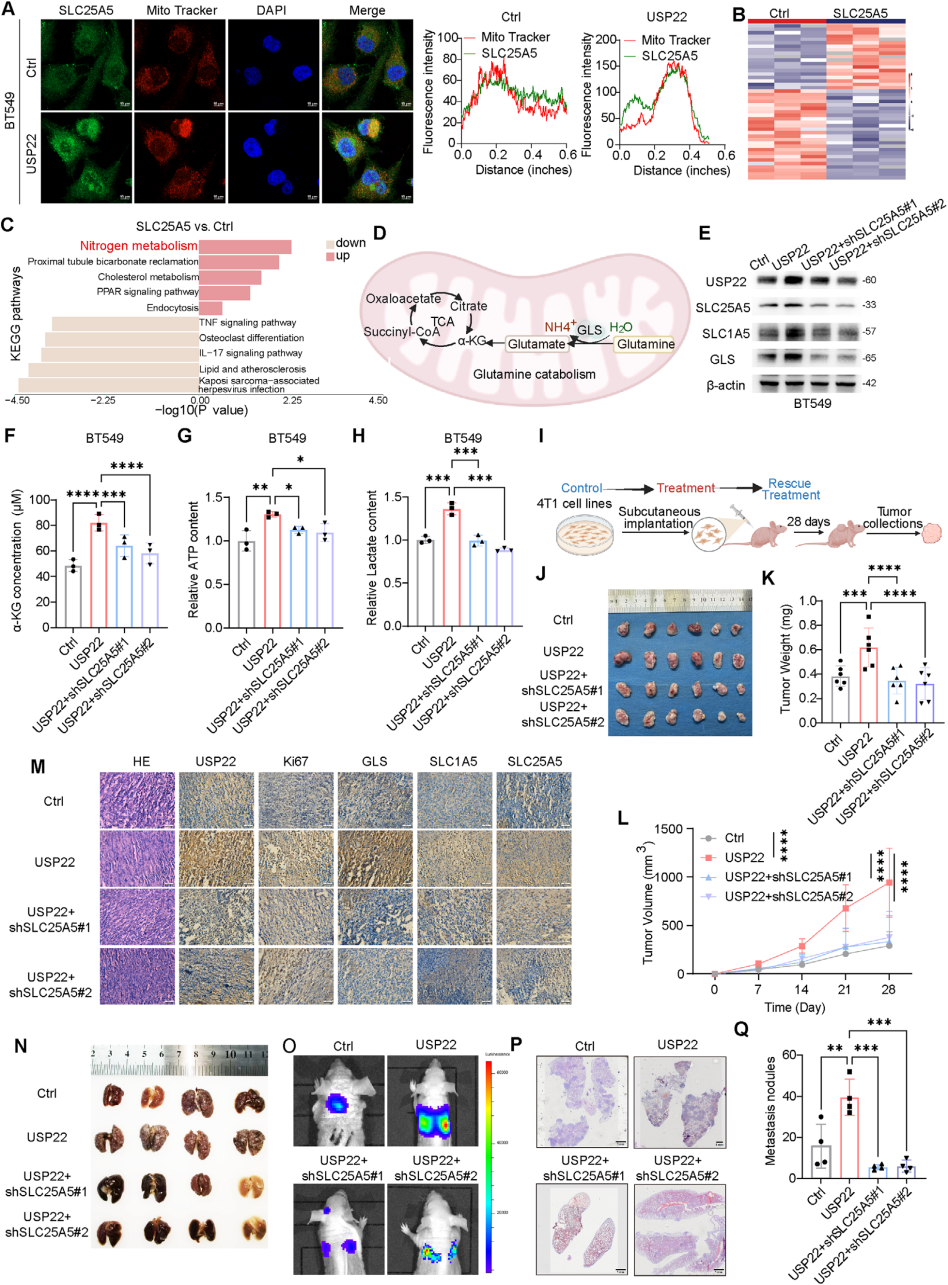
USP22 promotes glutamine metabolism of TNBC cells by upregulating SLC25A5. (A) Subcellular localization of SLC25A5 in control and USP22‐overexpressing BT549 cells. Scale bars, 10 µm. (B) Proteins related to SLC25A5 identified through proteomics analysis. (C) KEGG enrichment identified the proteins. (D) Glutamine catabolism. (E) USP22 was overexpressed in BT549 cells, and SLC25A5 was knocked down using two shRNAs. The expression levels of related proteins. (F–I) α‐KG content (F), ATP level (G), and lactate content (H) of BT549 cells. (I) Subcutaneous xenograft tumor model. (J–L) Tumor images (J), weights (K), and volumes (L) of 4T1‐derived xenograft tumors (*n* = 6). (M) IHC staining for USP22, Ki‐67, GLS, SLC1A5, and SLC25A5 in subcutaneous tumors. Scale bars, 50 µm. (N) Pulmonary metastases in nude mice after tail vein injection of 4T1 cells. (O) Bioluminescence signal intensities. (P) Hematoxylin and eosin staining images of lung tissues. (Q) Quantification of lung metastatic nodules. Statistical significance was determined by one‐way ANOVA (F–H,K,Q) or two‐way ANOVA (L). Data are expressed as means ± SD. ^*^ *p* < 0.05; ^**^ *p* < 0.01; ^***^ *p* < 0.001; ^****^ *p* < 0.0001.

### 3.9 Synergistic Effects of Peptide Inhibitors Targeting USP22 K134 Lactylation and CB‐839 in TNBC

Inhibition of GLS significantly inhibits tumor growth and disease progression [28]. Several GLS inhibitors demonstrate tumor‐specific antiproliferative effects against cancers [29]. USP22 promotes breast cancer growth and metastasis by regulating glutamine metabolism, providing a theoretical foundation for the synergistic therapeutic strategy combining USP22 inhibitor K134‐pe with CB‐839. K134‐pe and CB‐839 exhibited dose‐dependent antiproliferative effects in BT20 and BT549 cells in vitro, with IC_50_ values determined by CCK‐8 assays (Figure S11A,B). SynergyFinder analysis revealed marked synergistic efficacy (Figure 7A). Synergistically reduced α‐KG, ATP, and lactate levels, along with diminished EdU proliferation and transwell invasion, further validated combinatorial effects (Figure 7B–F). In vivo, K134‐pe monotherapy achieved tumor suppression comparable to that of CB‐839, while combination therapy induced significant tumor volume reduction in subcutaneous xenograft models (Figure 7G–K). Therefore, targeting USP22 K134 lactylation with K134‐pe synergistically enhances CB‐839 efficacy, suppressing TNBC growth and metastasis through dual‐pathway inhibition.

**FIGURE 7:**
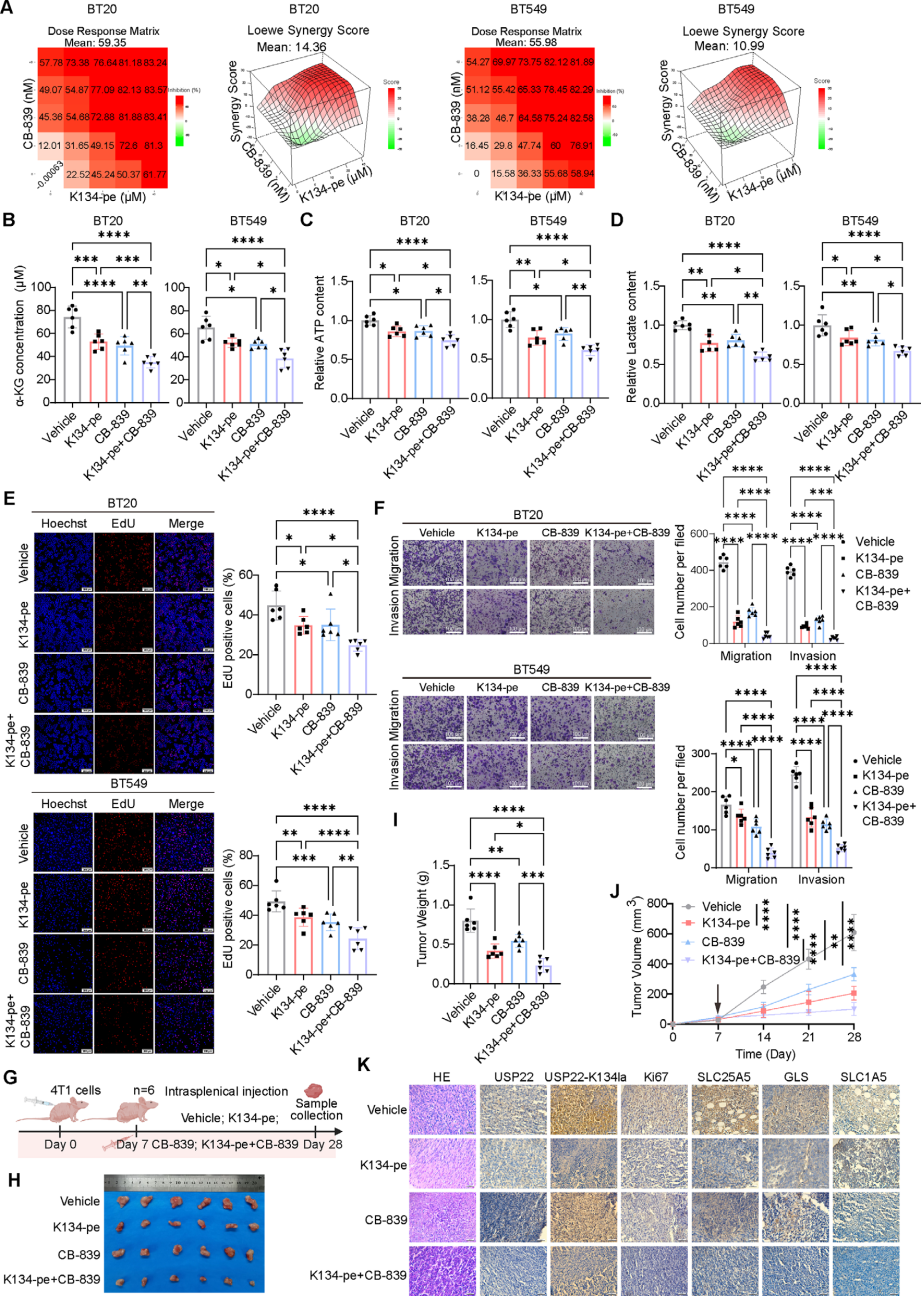
K134‐pe can synergize with CB‐839 to suppress the viability of TNBC cells. (A) Cells were pretreated with concentrations of K134‐pe, and then treated with doses of CB‐839 for 72 h. A CCK‐8 assay was then conducted. The cell viability data were plotted using the SynergyFinder software, and the Loewe synergistic effect score was calculated. A score >10 was considered a strong synergistic effect. (B–D) α‐KG content (B), ATP level (C), and lactate content (D) of BT20 and BT549 treated with K134‐pe (10 µm), CB‐839 (10 nm), or a combination for 24 h. (E,F) EdU assays and transwell assays of BT20 and BT549 cells treated with K134‐pe (10 µm), CB‐839 (10 nm), or a combination for 24 h. Scale bars, 200 µm (E). Scale bars, 100 µm (F). (G) Schematic representation of the subcutaneous xenograft tumor model. (H–J) Tumor images (H), weights (I), and volumes (J) monitored during the treatment (*n* = 6). (K) IHC staining. Scale bars, 50 µm. Statistical significance was determined using a one‐way ANOVA test. Data are expressed as means ± SD. ^*^ *p* < 0.05; ^**^ *p* < 0.01; ^***^ *p* < 0.001; ^****^ *p* < 0.0001.

### 3.10 GPR91 Senses α‐KG to Mediate Tumor‐Macrophage Interactions

Tumor‐derived α‐KG activates macrophages into functional TAMs and accelerates tumor progression [8, 30]. α‐KG regulates carbon‐nitrogen metabolism and amino acid homeostasis and acts as a ligand for G protein‐coupled receptors [31]. While G protein‐coupled receptor 91 (GPR91) mediates succinate‐induced M2 polarization [32], USP22 knockdown did not significantly alter succinate levels but markedly reduced α‐KG production (Figure 1D). We hypothesize that GPR91 may drive M2 polarization in TNBC by sensing tumor microenvironmental α‐KG.

GPR91 expression was highest in macrophages and minimal in TNBC cells (Figure 8A). Following induction of macrophage polarization and conditioned medium treatment (Figure 8B,C), we observed upregulated expression of GPR91 and the M2 macrophage marker in macrophages, and this effect was positively regulated by USP22 (Figure 8D,E and Figure S12A). CD206 and CD86 staining showed that after coculture, the macrophages transformed into the typical M2 morphology, characterized by elongated and dispersed cells with high CD206 expression and low CD86 expression (Figure 8F). CB‐839 treatment of tumor cells leads to a CM that impairs macrophage M2 polarization, exogenous α‐KG alone promoted M2 polarization (Figure 8G). Moreover, GPR91 knockdown via siRNA, specifically in macrophages, prevented tumor‐induced M2 polarization, whereas overexpression of GPR91 in macrophages enhanced M2 polarization and further amplified α‐KG‐induced M2 polarization (Figure 8H). Although α‐KG also serves as a cofactor for TET/JMJC enzymes, and TET/JMJD3‐mediated reprogramming has been implicated in M2 polarization, our data indicate that α‐KG‐driven M2 polarization in the tumor microenvironment depends primarily on GPR91 signaling.

**FIGURE 8:**
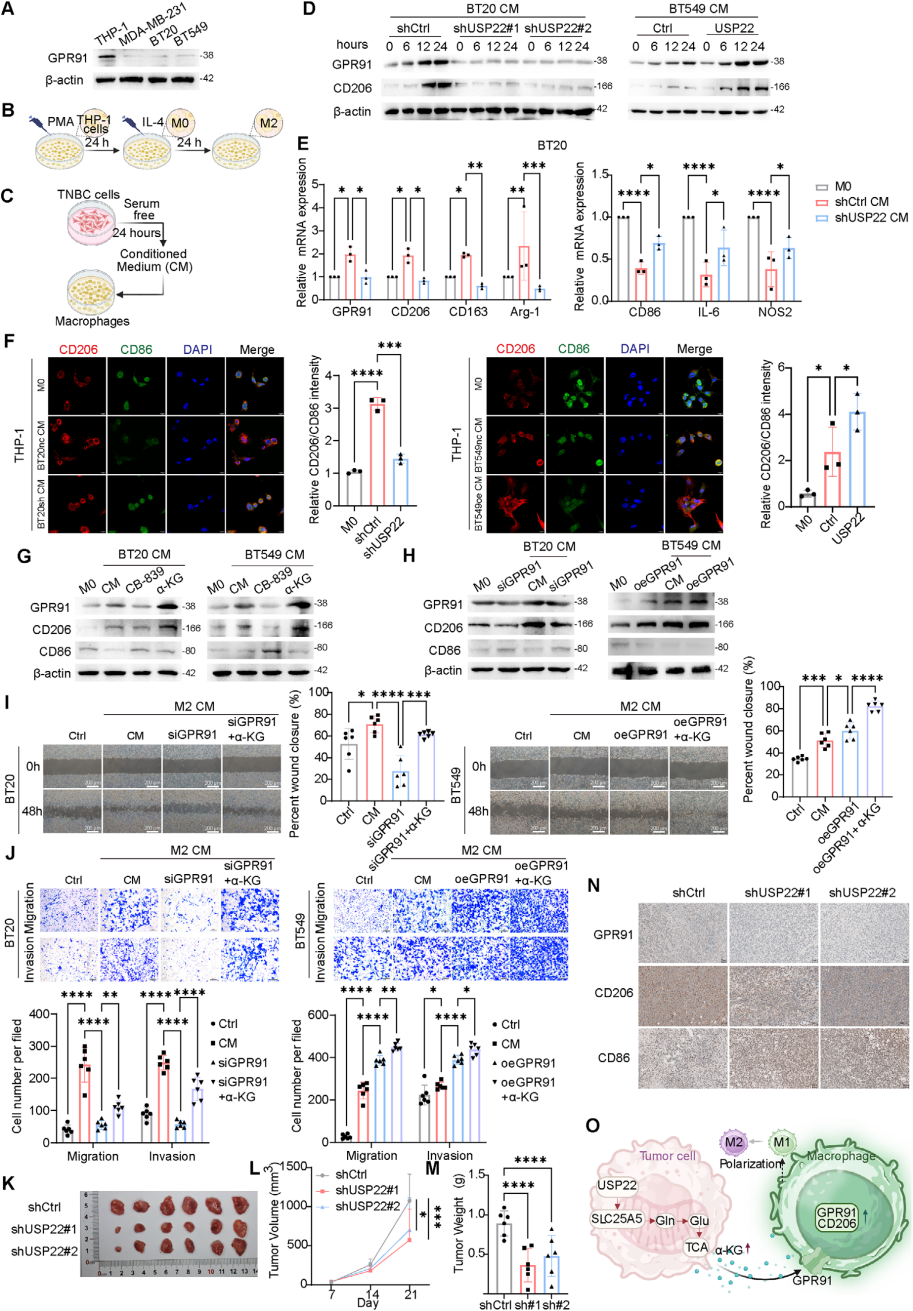
α‐KG stimulates GPR91 to drive M2 macrophage polarization. (A) Analysis of GPR91 in THP‐1, BT549, MDA‐MB‐231, and BT20 cells. (B) M0, M1, and M2 generation. (C) Schematic of CM collection from TNBC cells. (D) Western blotting for GPR91 and CD206 in M0 macrophages after treatment with the indicated tumor cell CM for 24 h. (E) Relative mRNA expression of M1/M2 markers in macrophages treated with M0, shCtrl CM, or shUSP22 CM. (F) IF of CD206 and CD86 in macrophages cocultured with BT20 or BT549 CM, with quantification of CD206/CD86 intensity. Scale bars, 25 µm. (G) After treating the M0 macrophages with the indicated tumor cell CM for 24 h, western blotting analysis was performed on the expression of GPR91, CD206, and CD86. (H) GPR91 was knocked down using siRNA in macrophages, and the cells were either treated with or without CM. Western blotting analysis of GPR91, CD206, and CD86 expression. (I) Wound healing assay of BT20 and BT549 cells treated with the indicated CM for 24 h. Scale bars, 200 µm. (J) Transwell assay of BT20 and BT549 cells treated with the indicated CM for 24 h. Scale bars, 100 µm. (K) Representative images of subcutaneous tumors formed by control and USP22‐knockdown MDA‐MB‐231 cells in BALB/c nude mice. (L) Tumor growth curves of control and USP22‐knockdown groups. (M) Tumor weights at the endpoint. (N) IHC staining for GPR91, CD206, and CD86 in subcutaneous tumors. Scale bars, 50 µm. (O) Schematic illustration. Statistical significance was determined by one‐way ANOVA (E, F, I, M) or two‐way ANOVA (J,L). Data are expressed as means ± SD. ^*^ *p* < 0.05; ^**^ *p* < 0.01; ^***^ *p* < 0.001; ^****^ *p* < 0.0001.

M2 macrophages promote tumor progression [33, 34]. We assessed their GPR91‐dependent impact on TNBC cell migration and invasion by detecting the effects of different pretreated macrophage CM on tumor cells. Compared with the untreated control group, the CM of GPR91‐knockdown macrophages significantly weakened the invasion and migration ability of TNBC cells. Conversely, GPR91 overexpression markedly enhanced invasion and migration, with further enhancement upon α‐KG addition (Figure 8I,J). Notably, in the absence of macrophages, α‐KG alone did not affect TNBC invasion or migration (Figure S12B). In vivo, using MDA‐MB‐231 cells in a BALB/c nude mouse subcutaneous tumor model, USP22 knockdown significantly reduced tumor growth (Figure 8K–M). IHC staining further revealed decreased GPR91 and CD206 expression, along with increased CD86 expression (Figure 8N). Therefore, USP22 may mediate glutamine‐derived α‐KG activation of GPR91 to induce M2 macrophage polarization, promoting TNBC invasion and metastasis (Figure 8O).

## 4 Discussion

Tip60 catalyzes the lactylation of USP22 at K134, simultaneously stabilizing USP22 protein levels and facilitating its mitochondrial location. Moreover, USP22 stabilizes the glutamine transporter SLC25A5 through its deubiquitinase activity. Restoring SLC25A5 expression in USP22‐knockdown cells effectively rescued the resultant metabolic dysregulation and poor survival. Clinically, increased expression levels of USP22 and SLC25A5 were significantly associated with a poor prognosis in patients with TNBC. Furthermore, USP22 regulates glutamine metabolism to promote α‐KG secretion, which activates M2 macrophages and suppresses the antitumor immune response. Using proteomics, metabolomics, and steady‐state fractional enrichment experiments, we revealed that the Tip60/USP22/SLC25A5 axis is a central regulator of glutamine reprogramming that promotes TNBC progression. These findings underscore the therapeutic potential of targeting this axis for TNBC treatment.

Dysregulation or reprogramming of metabolic processes drives the occurrence and development of malignant tumors [35]. Furthermore, abnormal glutamine metabolism is a common feature of various cancers [36], and rapidly proliferating tumor cells use glutamine as a source of carbon and nitrogen to produce vital biomolecules. The potential of glutamine metabolism as a therapeutic target is therefore becoming increasingly evident [37]. Strategies targeting cancer glutamine metabolism focus on inhibiting GLS or glutamine transporters [38]. For instance, silencing SLC1A5 or GLS in tumor cells effectively inhibits tumor growth in vivo [39, 40].

USP22 promotes oncogenesis in various cancers [41, 42, 43, 44], regulates protein stability through its deubiquitinating enzyme activity, and affects multiple signaling pathways. This plays a key regulatory role in cell growth, differentiation, and apoptosis. USP22 primarily catalyzes the deubiquitination of histones H2A and H2B, facilitating gene transcription activation [45]. USP22 also plays an important oncogenic role in TNBC. As a key regulatory factor, it drives tumor metabolic reprogramming and provides a metabolic basis for tumor growth, metastasis, and drug resistance [20, 46]. However, the core target genes and signaling pathways through which USP22 regulates glutamine metabolism remain unclear. Here, we revealed a positive feedback pathway centered on Tip60/USP22/SLC25A5 that drives glutamine metabolic reprogramming and tumor progression in TNBC.

TNBC is more dependent on glutamine than other subtypes of breast cancer. Additionally, USP22 promotes TNBC progression by driving glutamine metabolism, providing biosynthetic precursors, and generating key metabolites, such as α‐KG and lactate. These metabolites supply energy and activate signal cascades. Glutamine‐derived lactate activates the lactyltransferase Tip60, which catalyzes the lactylation modification of USP22 K134, thereby stabilizing the USP22 protein and enhancing its function.

The modified USP22 was transported to the mitochondria, where it interacted with SLC25A5, an amino acid transporter responsible for mitochondrial glutamine input, located in the inner mitochondrial membrane [47]. SLC25A5 significantly promoted the proliferation, migration, and invasion of TNBC cells in vitro, and was a key effector molecule downstream of USP22. USP22, which is localized to the mitochondria, stabilized the SLC25A5 protein through deubiquitination modification, preventing degradation and ensuring a continuous supply of substrates for glutamine catabolism. SLC25A5 is indispensable for enhancing USP22‐mediated glutamine metabolism and tumor‐promoting effects; its absence can completely block metabolic reprogramming and the tumor‐promoting phenotype induced by USP22 overexpression.

We designed a cell‐penetrating peptide that specifically inhibits USP22 lactylation at K134. When combined with the GLS inhibitor CB‐839, a significant synergistic effect was observed in TNBC. These results provide a theoretical basis for combined therapy targeting both the upstream lactylation modification and the downstream metabolic enzymes dependent on SLC25A5 substrate supply.

Macrophages are important immune cells in the tumor microenvironment that play a significant role in metastasis. Tumor cells regulate the functions and phenotypes of macrophages by secreting metabolites, metabolic enzymes, and extracellular vesicles [48, 49]. Similarly, macrophages can regulate the malignant biological phenotypes of tumor cells, such as proliferation and invasion, by secreting soluble factors [50]. Therefore, the interplay between tumor cells and macrophages warrants further investigation. We used metabolomics and steady‐state fractional enrichment analysis to discover that USP22 increases glutamine metabolism in TNBC cells, resulting in higher levels of α‐KG and lactate. Furthermore, α‐KG, derived from glutamine in tumor cells, activates the GPR91 receptor on macrophages, promoting the alternative‐activated macrophage (M2) phenotype. Conversely, GPR91‐activated macrophages further promote tumor cell migration and invasion, forming a positive feedback loop. Overall, GPR91 mediates the interaction between tumor cells and macrophages during TNBC metastasis.

Several limitations of this study should be acknowledged. First, although our findings reveal an important role for USP22 K134 lactylation in TNBC progression, validation in human TNBC clinical samples remains limited, and larger independent cohorts are needed to confirm its clinical relevance and prognostic value. Second, the precise molecular mechanism underlying USP22 mitochondrial translocation has not been fully elucidated, and further structural and biochemical studies are required to define the key regulatory steps and interacting partners. Third, whether this mechanism is universal across other tumor types remains to be determined, as our study focused primarily on TNBC. Fourth, the in vivo functional validation of macrophages needs to be further improved; future studies using macrophage‐specific genetic manipulation, such as conditional knockout or depletion in orthotopic tumor models, will be important to clarify the contribution of macrophages to USP22 K134 lactylation‐mediated tumor progression. These limitations do not diminish the core conclusions of this study but provide clear directions for future investigation.

In summary, this study suggests that USP22 regulates glutamine metabolism in TNBC. Glutamine‐derived lactate undergoes K134 lactylation modification, which is catalyzed by Tip60. This activates USP22 and causes it to translocate to the mitochondria. USP22 stabilizes the glutamine transporter SLC25A5, enhances glutamine uptake, and forms a positive feedback loop that drives tumor proliferation and invasion. Moreover, α‐KG activates the GPR91 receptor on macrophages, inducing M2 polarization and promoting TNBC metastasis. Targeting USP22 lactylation and glutamine metabolism (CB‐839) has a synergistic antitumor effect, offering a new therapeutic strategy for TNBC treatment. However, the existence of other USP22 PTMs in TNBC requires further exploration.

## Author Contributions

**Fang Zhen**, **Hongyi Wang**, and **Jing Hu** designed the research. Fang Zhen and Hongyi Wang carried out experiments. **Ningzhi Zhang**, **Le Wang**, **Huiyuan Tian**, **Luxi Kang**, and **Zhanpeng Liu** provided support with data analysis. Jing Hu wrote the paper and critically reviewed the manuscript. All authors read and approved the final manuscript.

## Funding

This work was supported by the National Natural Science Foundation of China grant (82372839 and 81972162 to J. Hu), and Climbing program of Harbin Medical University Cancer Hospital (No. PDYS2024‐11 to J. Hu).

## Conflicts of Interest

The authors declare no conflicts of interest.

## Supporting information




**Supporting File 1**: advs78486‐sup‐0001‐SuppMat.docx.

**Supporting File 2**: advs78486‐sup‐0002‐SuppMat.jpg.

**Supporting File 3**: advs78486‐sup‐0003‐SuppMat.jpg.

## Data Availability

The data that support the findings of this study are available from the corresponding author upon reasonable request.
