# Supplementary material for: Tip60‐Mediated USP22 Lactylation Drives Glutamine Metabolic Reprogramming via SLC25A5 in TNBC
Source: Adv Sci (Weinh). 2026 Oct 26:e78486. Online ahead of print. doi: 10.1002/advs.78486 (PMC13710710; doi:10.1002/advs.78486)
Supplement: Supplementary file 1 [file ADVS-9999-e78486-s002.docx]

Supplementary Materials for

Tip60-mediated USP22 lactylation drives glutamine metabolic reprogramming via SLC25A5 in TNBC

Fang Zhen et al.

Corresponding author: Jing Hu, [hujing@ems.hrbmu.edu.cn](mailto:hujing@ems.hrbmu.edu.cn)

**Figure S1. Glutamine addiction in TNBC.** (**A**) Glutamine metabolism levels in tumor and normal breast tissues analyzed using the GEO database (GSE45827). (**B**) Differences in glutamine metabolism across breast cancer molecular subtypes (GSE45827). (**C**) Kaplan–Meier survival analysis stratified by glutamine metabolism activity and risk score (*P* = 0·036) (GSE58812). (**D**) Colony formation assay showing BT20 (TNBC) cells were markedly more sensitive to glutamine deprivation than T47D (luminal) and SKBR3 (HER2+) cells. Proliferative ability of TNBC cells treated with glutamine deprivation was examined using CCK-8 (**E**) and EdU incorporation assays (**F**). Scale bars, 200 μm. (**G**) Colony formation ability of TNBC cells treated with glutamine deprivation was detected using a colony formation assay. (**H**) The cell migration ability of TNBC cells treated with glutamine deprivation was detected by wound-healing assays. Scale bars, 200 μm. (**I**) The cell migration and invasion ability of TNBC cells treated with glutamine deprivation was detected by transwell migration assays. Scale bars, 100 μm. Statistical significance was determined using a one-way ANOVA test or an unpaired Student’s t-test. Data are expressed as means ± SD. **P* < 0.05; ***P* < 0.01; ****P* < 0.001; *****P* < 0.0001; ns, no statistical significance.

**Figure S2. USP22 promotes TNBC cell proliferation.** (**A**) Western blotting was used to verify the efficiency of USP22 knockdown and overexpression in TNBC cell lines. (**B**) qRT-PCR was used to validate the efficiency of USP22 knockdown and overexpression at the mRNA level. (**C, D**) The cell proliferative capacity after USP22 knockdown or overexpression was detected by CCK-8 assay and EdU staining assay. Scale bars, 200 μm. (**E**) Colony formation ability after USP22 knockdown or overexpression was detected using a colony formation assay. Statistical significance was determined by a one-way ANOVA test or an unpaired Student’s t-test. Data are expressed as means ± SD. **P* < 0.05; ***P* < 0.01; ****P* < 0.001; *****P* < 0.0001; ns, no statistical significance.

**Figure S3. USP22 migration and invasion of TNBC.** (**A**) The wound-healing assay showed the effect of USP22 knockdown and overexpression on the migration ability of TNBC cells. Quantitative and statistical analyses were performed according to the wound area to reflect cell migration ability. Scale bars, 200 μm. (**B**) Transwell assay was used to evaluate the effect of USP22 knockdown and overexpression on TNBC cell migration and invasion. Scale bars, 100 μm. (**C**) Pulmonary metastases in nude mice after tail vein injection of shCtrl or shUSP22 4T1 cells. (**D**) Bioluminescence signal intensities in the shCtrl and shUSP22 groups. (**E**) Representative H&E staining images of lung tissues. Scale bars, 1000 μm. (**F**) Quantification of lung metastatic nodules. Statistical significance was determined using one-way ANOVA test for A and F; and two-way ANOVA test for B. Data are expressed as means ± SD. **P* < 0.05; ***P* < 0.01; ****P* < 0.001; *****P* < 0.0001.

**Figure S4. USP22 mediates glutamine metabolism in TNBC.** (**A**) Glutamine concentration in cell culture supernatants. (**B**) Glutamine uptake. (**C-G**) Levels of intracellular glutamate (**C**), α-KG (**D**), and enzymatic activities of GLS (**E**), GLUD1 (**F**), and GOT (**G**) in TNBC cells following USP22 knockdown or overexpression. (**H**) Seahorse ECAR analysis to evaluate glycolytic phenotype. (**I**) ATP levels. (**J**) Lactate levels. Statistical significance was determined by a one-way ANOVA test or an unpaired Student’s t-test. Data are expressed as means ± SD. **P* < 0.05; ***P* < 0.01; ****P* < 0.001; *****P* < 0.0001.

**Figure S5. Lactate is upregulated in TNBC.** (**A**) Differences in lactate and USP22 between high and low glutamine risk groups (GSE45827). (**B**) Diagram of TNBC tissue patterns. (**C, D**) IHC and IF staining with Pan-Kla antibody in postoperative tumor specimens and adjacent tissues of TNBC. Scale bars, 50 μm. (**E**) Correlation analysis between USP22 score and Pan-Kla score in TNBC samples (n=50). (**F**) IF staining to analyze the expression of Pan-Kla in xenograft tumors. Scale bars, 50 μm. Statistical significance was determined by a one-way ANOVA test (F) or an unpaired Student’s t-test (C). Data are expressed as means ± SD. **P* < 0.05; ***P* < 0.01; ****P* < 0.001.

**Figure S6. Targeting USP22 K134 lactylation inhibits tumor progression.** (**A**) Seahorse ECAR analysis in BT549 cells treated with Vehicle, K134-peptide, or K134R-peptide. (**B-D**) ATP (B), lactate (C), and levels of α-KG (D) in BT549 cells treated with K134-pe or K134R-pe (10 μM) for 24 h. (**E, F**) The cell proliferative capacity after treatment with K134-pe or K134R-pe was detected by CCK-8 and EdU staining assays. Scale bars, 200 μm. (**G, H**) Wound-healing and transwell migration assays were performed on BT20 and BT549 cells treated with K134-pe or K134R-pe (10 μM) for 24 h. Scale bars, 200 μm (G). Scale bars, 100 μm (H). Statistical significance was determined by one-way ANOVA (B–D, F, G) or two-way ANOVA (A, E, H). Data are expressed as means ± SD. **P* < 0.05; ***P* < 0.01; ****P* < 0.001; *****P* < 0.0001; ns, no statistical significance.

**Figure S7. SLC25A5 promotes TNBC proliferation, migration, and invasion.** **(A, B)** Efficiency of SLC25A5 knockdown and overexpression was confirmed at both the protein (western blotting) and mRNA (qRT-PCR) levels in TNBC cell lines. (**C, D**) The cell proliferative capacity after SLC25A5 knockdown or overexpression was assessed using CCK-8 and EdU staining assay. Scale bars, 200 μm. (**E**) Colony formation ability was evaluated after SLC25A5 knockdown or overexpression. (**F**) Wound-healing assays demonstrated the impact of SLC25A5 knockdown and overexpression on TNBC cell migration. Migration ability was quantified based on wound area reduction. Scale bars, 200 μm. (**G**) Transwell assays evaluated the effects of SLC25A5 knockdown and overexpression on TNBC cell migration and invasion. Scale bars, 100 μm. （**H**） Glutamine uptake in BT20 and BT549 cells after SLC25A5 knockdown or overexpression. (**I**) Mitochondrial glutamate content . (**J**) α-KG concentration. Statistical significance was determined by one-way ANOVA, two-way ANOVA, or an unpaired two-tailed Student’s t-test. Data are expressed as means ± SD. **P* < 0.05; ***P* < 0.01; ****P* < 0.001; *****P* < 0.0001; ns, no statistical significance.

**Figure S8. Tip60 induces lactylation of USP22 to strengthen SLC25A5 stability.** (**A**) Western blotting analysis of total lysates and anti-SLC25A5 IP of BT20 cells that were transfected with Flag-USP22 and His-Tip60 constructs and treated with MG132 (10 μM) for 6 hours. (**B**) Western blotting analysis of BT20 cell total lysates and anti-SLC25A5 IP, as indicated, from cells transfected with Flag-USP22 WT or K134R constructs and treated with MG132 (10 μM) for 6 hours. (**C**) BT20 cells were treated with 200 μg/ml CHX with or without His-Tip60, collected at designated time points, and subsequently subjected to western blotting analysis. (**D**) BT20 cells were transfected with either USP22 or USP22 K134R and then treated with 200 μg/ml CHX. The cells were collected at the indicated times and subjected to Western blotting analysis.

**Figure S9. The expression of USP22 is positively correlated with the expression of SLC25A5 in TNBC.** (**A**) Schematic diagram. (**B**) Western blotting analysis of USP22 and SLC25A5 in TNBC tissue samples. (**C**) Representative images of the IHC staining of USP22 and SLC25A5 in TNBC tissues (n = 50). Scale bars, 50 μm. (**D-F**) Quantitative analysis of USP22 (D), SLC25A5 (E) and USP22 K134la (F) IHC staining. (**G**) Correlation between USP22 and SLC25A5 expression in TNBC tissues (Pearson correlation coefficient, R). (**H, I**) Correlation of USP22-K134la score with USP22 score and SLC25A5 score. (**J**) Analysis of the correlation between USP22 and SLC25A5 expression in TNBC using the TIMER database. (**K-M**) GEPIA2 database analysis of overall survival in TNBC patients: Kaplan–Meier curves for USP22 (K), SLC25A5 (L), and co-expression (M). (**N-P**) Analysis of USP22 and SLC25A5 levels in high- and low-risk glutamine metabolism groups using GEO data (GSE65194, GSE76275, GSE103091). (**Q-S**) Kaplan–Meier curves for the overall survival of TNBC patients based on GEO data: USP22 (Q), SLC25A5 (R), and combined expression (S) groups (GSE58812). Statistical significance was determined by unpaired Student’s t-test (D, E, F), Pearson correlation (G-I), log-rank test (K-M, Q-S), and one-way ANOVA (N-P). Data are expressed as means ± SD. **P* < 0.05; ***P* < 0.01; ****P* < 0.001; *****P* < 0.0001; ns, no statistical significance.

**Figure S10. USP22 promotes tumor progression by regulating SLC25A5.** (**A**) IF staining analyzed the subcellular localization of SLC25A5 in BT20 cells. Scale bars, 10 μm. (**B, C**) Transwell migration assays were performed on BT549 and BT20 cells. The rescue experiments for SLC25A5 knockdown were performed by USP22 overexpression in BT549 cells. The rescue experiments for SLC25A5 overexpression were performed by USP22 knockdown in BT20 cells. Scale bars, 100 μm. (**D**) USP22 was knocked down in BT20 cells, and SLC25A5 was overexpressed. The expression levels of related proteins were detected by Western blotting analysis. (**E-G**) α-KG content (E), ATP level (F), and lactate content (G) of BT20 cells. Statistical significance was determined by a one-way ANOVA test (E-G) and two-way ANOVA test for B and C. Data are expressed as means ± SD. **P* < 0.05; ***P* < 0.01; ****P* < 0.001; *****P* < 0.0001.

**Figure S11. Determination of drug IC50 values.** Dose-response curves show the inhibitory effect of K134-pe (**A**) and CB-839 (**B**) on the growth of BT20 and BT549 cells.

**Figure S12. α-KG stimulates GPR91 to drive M2 macrophage polarization.** (**A**) Relative mRNA expression of M2 markers and M1 markers in macrophages treated with M0, Ctrl CM, or USP22 CM from BT549 cells. (**B**) Transwell migration and invasion assays in BT20 and BT549 cells treated with or without α-KG. Scale bars, 50 μm. Statistical significance was determined using an one-way ANOVA test. Data are expressed as means ± SD. **P* < 0.05; ***P* < 0.01; ****P* < 0.001; *****P* < 0.0001; ns, no statistical significance.

**Supplemental Table 1. Information of target sequences used in the study**

| **siRNAs** | sense（5′-3′） |
| --- | --- |
| sicontrol | TTCTCCGAACGTGTCACGT |
| siUSP22#1 | CACGGACAGTCTCAACAAT |
| siUSP22#2 | CTGCAAAGGTGATGACAAT |
| siEP300 | GCUACUGAAGAUAGAUUAATT |
| siGCN5 | CGAUGUUCGAGCUCUCAAATT |
| siPCAF | GCAGAAGGAGAUAAUUAAATT |
| siPCBP4 | GGCGAGACUGUAAAGCGAATT |
| siTip60 | GGACAGCUCUGAUGGAAUATT |
| siGPR91 | GGAGACGUGCUCUGCAUAATT |
| **shRNAs** | sense（5′-3′） |
| shControl | GATCCGTTCTCCGAACGTGTCACGTAATTCAAGAGATTACGTGACACGTTCGGAGAATTTTTTC |
| shUSP22#1 | GATCCGCACGGACAGTCTCAACAATTTCAAGAGAATTGTTGAGACTGTCCGTGTTTTTTC |
| shUSP22#2 | GATCCGCTGCAAAGGTGATGACAATTTCAAGAGAATTGTCATCACCTTTGCAGTTTTTTC |
| shUSP22#3 | GATCCGCATGCTTCATGAACTGCATCGTGCATTCAAGAGATGCACGATGCAGTTCATGAAGCATGTTTTTTC |
| shSLC25A5#1 | GATCACTGCAGATAAGCAATA |
| shSLC25A5#2 | GGTGGTGTGGACAAGAGAACC |
| shSLC25A5#3 | GTGTCTGTGCAGGGTATTATC |

**Supplementary Table 2. Information of primer sequences used in the study**

| Symbol | Primer | Sequence (5’–3’) |
| --- | --- | --- |
| USP22 | Forward | GGTCCCCTCACATCCCGTATA |
|  | Reverse | TCTTCCCATTGTCATCACCTTTG |
| SLC25A5 | Forward | CGTGTCTGTGCAGGGTATTAT |
|  | Reverse | CTGATGACGATGTGAGTGTTCT |
| GPR91 | Forward | GTGTCATCAACCCTGTCTTCT |
|  | Reverse | GCCCATCTGCTAAAGGATGTA |
| CD163 | Forward | GGGATGTCCAACTGCTATCAA |
|  | Reverse | GACTCATTCCCACGACAAGAA |
| ARG1 | Forward | CAGGGCTACTCTCAGGATTAGA |
|  | Reverse | CCGAAACAAGCCAAGGTTATTG |
| NOS2 | Forward | GTCAGAGTCACTATCCTCTTTG |
|  | Reverse | GCAGCTCAGCCIGTACTTATC |
| IL6 | Forward | CCAGGAGAAGATTCCAAAGATGTA |
|  | Reverse | CGTCGAGGATGTACCGAATTT |
| CD86 | Forward | CCAGACCACATTCCTTGGATTA |
|  | Reverse | TCTTCCCTCTCCATTGTGTTG |
| β-actin | Forward | TCCATCATGAAGTGTGACGT |
|  | Reverse | GAGCAATGATCTTGATCTTCAI |
